# OsCNGC13 promotes seed-setting rate by facilitating pollen tube growth in stylar tissues

**DOI:** 10.1371/journal.pgen.1006906

**Published:** 2017-07-14

**Authors:** Yang Xu, Jie Yang, Yihua Wang, Jiachang Wang, Yang Yu, Yu Long, Yunlong Wang, Huan Zhang, Yulong Ren, Jun Chen, Ying Wang, Xin Zhang, Xiuping Guo, Fuqing Wu, Shanshan Zhu, Qibing Lin, Ling Jiang, Chuanyin Wu, Haiyang Wang, Jianmin Wan

**Affiliations:** 1 State Key Laboratory for Crop Genetics and Germplasm Enhancement, Jiangsu Plant Gene Engineering Research Center, Nanjing Agricultural University, Nanjing, China; 2 National Key Facility for Crop Gene Resources and Genetic Improvement, Institute of Crop Science, Chinese Academy of Agricultural Sciences, Beijing, China; 3 Institute of Food Crops, Jiangsu Academy of Agricultural Sciences, Nanjing, China; 4 State Key Laboratory of Plant Physiology and Biochemistry, College of Biological Sciences, National Plant Gene Research Centre (Beijing), China Agricultural University, Beijing, China; University of Arizona, UNITED STATES

## Abstract

Seed-setting rate is a critical determinant of grain yield in rice (*Oryza sativa* L.). Rapid and healthy pollen tube growth in the style is required for high seed-setting rate. The molecular mechanisms governing this process remain largely unknown. In this study, we isolate a dominant low seed-setting rate rice mutant, *sss1-D*. Cellular examination results show that pollen tube growth is blocked in about half of the mutant styles. Molecular cloning and functional assays reveals that *SSS1-D* encodes OsCNGC13, a member of the cyclic nucleotide-gated channel family. *OsCNGC13* is preferentially expressed in the pistils and its expression is dramatically reduced in the heterozygous plant, suggesting a haploinsufficiency nature for the dominant mutant phenotype. We show that OsCNGC13 is permeable to Ca^2+^. Consistent with this, accumulation of cytoplasmic calcium concentration ([Ca^2+^]_cyt_) is defective in the *sss1-D* mutant style after pollination. Further, the *sss1-D* mutant has altered extracellular matrix (ECM) components and delayed cell death in the style transmission tract (STT). Based on these results, we propose that OsCNGC13 acts as a novel maternal sporophytic factor required for stylar [Ca^2+^]_cyt_ accumulation, ECM components modification and STT cell death, thus facilitating the penetration of pollen tube in the style for successful double fertilization and seed-setting in rice.

## Author summary

Rice is not only the staple food for more than half of the world’s population, but also a model species for plant developmental and genetic studies. After pollination, rice pollen grains adhere and hydrate at the surface of stigmatic papilla cells. Then, the germinated pollen tubes invade the stigma and navigate through the style transmission tract to reach the micropyle of the embryo sac for fertilization. During this long and arduous process, pollen tube requires abundant communication with the surrounding sporophytic maternal tissues. However, how the growth of pollen tube is regulated by maternal tissue remains largely elusive. This work identifies a typical cyclic nucleotide-gated channel protein in rice, OsCNGC13, which can mediate Ca^2+^ inward current. Our results suggest that OsCNGC13 acts as a novel maternal sporophytic factor required for stylar [Ca^2+^]_cyt_ accumulation, extracellular matrix components modification and style cell death, thus facilitating the penetration of pollen tube in the style for successful double fertilization and seed-setting in rice. These findings provide new insights into the molecular genetic control mechanisms of seed-setting rate/grain yield in rice and expand our knowledge on the cyclic nucleotide-gated channel proteins in plant sexual reproduction.

## Introduction

Rice (*Oryza sativa* L.) is not only the staple food for more than half of the world’s population, but also a model species for plant developmental and genetic studies [1]. Panicle numbers, grain number per panicle, grain weight and seed-setting rate constitute the major determinants of grain yield in rice [2]. During the past few decades, great progress has been made in elucidating the molecular genetic control mechanisms of panicle numbers, grain number per panicle and grain weight in rice [2]. Recently, increasing effort has been made to elucidate the genetic control of seed-setting rate in rice as low seed-setting rate of *Indica-Japonica* hybrid has become a bottleneck limiting further improvement of hybrid grain yield [3].

Low seed-setting rate in rice could result from spikelet sterility due to abnormal floret structures, defective pollen grain or embryo sac development, impaired anther dehiscence, gametophytic incompatibility or inappropriate temperature at the reproductive stage. Several genes underlying seed-setting rate have been characterized. For examples, *Pollen semi*-*sterility1* encodes a kinesin-1-like protein important for male meiosis, anther dehiscence, and fertility in rice [4]. *WA352* is a mitochondrial gene that confers wild abortive cytoplasmic male sterility in rice. WA352 inhibits the known nuclear-encoded mitochondrial protein COX11 functioning in peroxide metabolism, thereby triggering premature tapetal programmed cell death (PCD) in the anther and consequent pollen abortion [5]. The *S5* locus contains three tightly linked genes that regulate *indica*-*japonica* hybrid fertility [6]. A recent study also showed that defective pollen tube growth in pistil could also cause reduced panicle fertility and seed setting [7]. Despite the tremendous progress, however, our understanding of the genetic control of seed-setting rate in rice still remains very fragmented.

A critical determining factor of seed-setting rate in flowering plants is the rapid and directional pollen tube growth in the style transmission tissue (STT) to deliver the male gametes to the ovary of the pistils for successful double fertilization, and this process involves intensive communication between the pollen tube and the surrounding maternal sporophytic tissues [8]. In particular, pharmacological and genetic studies have shown that Ca^2+^, composition of the extracellular matrix (ECM) and PCD of STT cells are all required for healthy growth of pollen tube in the style [9–13]. However, the functional relationships among these factors in controlling pollen tube growth and reproduction are still poorly understood.

The cyclic nucleotide-gated channel proteins (CNGCs) were nonspecific, Ca^2+^-permeable cation channels and were first identified in vertebrate photoreceptors [14]. There are 20 members of CNGCs in Arabidopsis that are differentially expressed in all tissues [15]. AtCNGC2/5/6/7/8/9/10/16/18 have been demonstrated to mediate Ca^2+^ currents [16–19], while AtCNGC1 and AtCNGC2 have been demonstrated to facilitate K^+^ or other monovalent cation fluxes [20]. A wide range of biological functions has been reported for CNGC proteins. For example, it has been reported that AtCNGC2, AtCNGC11/12, AtCNGC11 and AtCNGC12 play a role in PCD [21–26]. AtCNGC16 was shown to be critical for stress tolerance in pollen reproductive development and AtCNGC14 was reported to regulate root gravitropism [27, 28]. Notably, the pollen tube tip plasma membrane-located protein AtCNGC18 was recently shown to be the long-sought essential Ca^2+^ channel for mediating external Ca^2+^ influx and pollen tube tip growth in Arabidopsis [18, 29]. In rice, 16 CNGC members were identified, and these genes are nominated according to their phylogenetic placement [30]. However, there is no reported functional studies on these rice CNGCs yet.

In this study, we isolated a dominant low seed-setting rate mutant in rice (*sss1-D*) and demonstrated that a mutation in a gene encoding cyclic nucleotide-gated channel (OsCNGC13) is responsible for the mutant phenotype. We found that *OsCNGC13* is preferentially expressed in the pistils, and that the mutation causes cytoplasmic calcium concentration ([Ca^2+^]_cyt_) loss, altered ECM components, and delayed cell death in the style. As a result, pollen tube growth is blocked in about half of the styles. Our results suggest that OsCNGC13 defines a novel maternal sporophytic factor that links stylar [Ca^2+^]_cyt_, ECM components modification and style cell death for promoting pollen tube growth and seed-setting in rice.

## Result

### *sss1-D* is a dominant low seed-setting rate rice mutant

**Fig 1:**
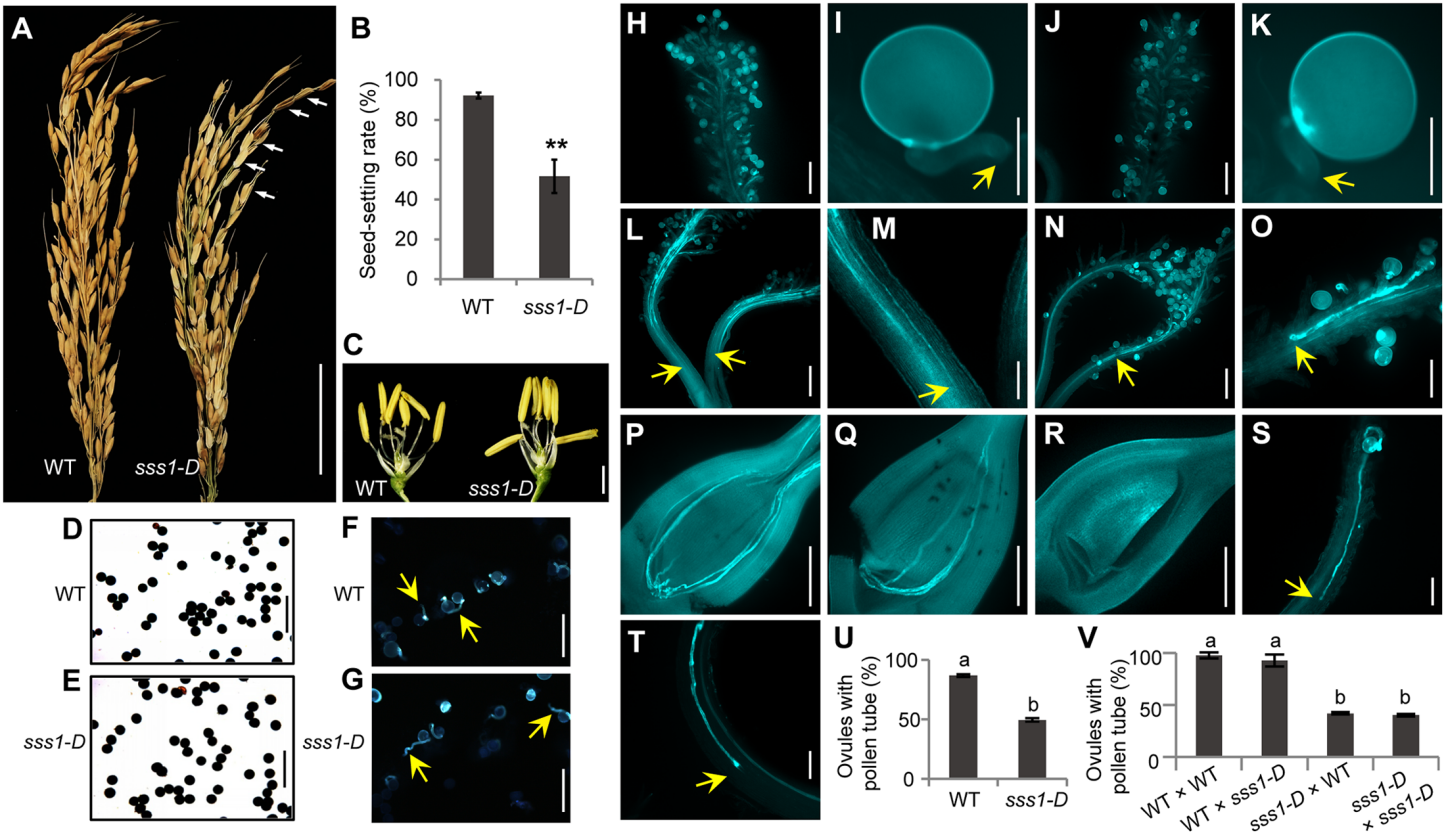
Pollen tube growth is impaired in *sss1-D*. (A) Comparison of mature panicle of wild type (WT) and *sss1-D*. Arrows indicate the sterile spikelets. (B) Seed-setting rate. Data are means ± SD (n > 5). **P<0.01 by the Student’s *t* test. (C) Floral structure of WT and *sss1-D*. (D and E) I_2_-KI staining of pollen grains of WT and *sss1*-*D*. (F and G) Aniline blue staining of the WT and *sss1*-*D* pollen germination in vitro. (H-K) Aniline blue staining showing normal germination of pollen grains on stigmas in selfed WT (H and I) and *sss1*-*D* (J and K) at 5 minutes after pollination (MAP). (L-O) Retardation of pollen tube growth in *sss1-D*. WT pollen tubes reach the bottom part of the style in most WT pistils at 30 MAP (L and M), whereas in some *sss1*-*D* pistils, pollen tubes stay at the stigma–style boundary (N and O). (P-V) Multiple pollen tubes can be observed in the WT ovule at 120 MAP (P), whereas the *sss1-D* ovule contains fewer or no pollen tubes (Q and R). Pollen tubes stay at the stigma–style boundary (S) or at the middle part of the style in majority of *sss1-D* pistils (T). (U) Quantification of ovaries with pollen tubes observed in the ovule at 120 MAP, showing defective pollen tube growth in more than half of *sss1-D* pistils. (V) Verification of the maternal effect of *sss1-D* for defective pollen tube growth. The *sss1-D* pollen tubes are capable of growing to ovule at 120 MAP in WT pistils, whereas the WT pollen tubes fail to reach the ovule in more than 50% of *sss1-D* pistils. Arrows point to pollen tubes in I and K, and pollen tube tips in L-O and S-T. Data in U and V are means ± SD from 3 replicates with > 30 pistils observed per replicate, and different letters indicate a significant difference at *P* < 0.01 by the Student’s *t*-test. Scale bars, 5 cm in (A); 2 cm in (C); 100 μm in (D-G,M,O,S,T); 300 μm in (H,J,L,N,P-R); 50 μm in (I and K).

In an effort to dissect the mechanism underling rice fertility, we isolated a dominant low seed-setting rate rice mutant named *semi-seed-setting rate1*-*Dominant* (*sss1-D*), by screening a ^60^Co-irradiated 9311 M_2_ population. Under normal field conditions, the seed-setting rate of the wild type (9311) was ~92%, while the mutant showed a much lower seed-setting rate (~51%) (Fig 1A and 1B). The floret structure, pollen fertility and pollen germination in vitro of the mutant appeared normal, compared to the wild type plants (Fig 1C–1G).

To determine the underlying cause of the fertility defect, we conducted reciprocal crosses between *sss1-D* and its wild type 9311. The results showed that when *sss1-D* was used as the pollen donor, the seed-setting rate was normal; however, when *sss1-D* was used as the pollen receiver, varying degrees of reduced seed-setting rates (ranging from 30% to 42%) were observed (S1 Table). This finding suggests that the low seed-setting rate of *sss1-D* is due to a maternal defect. Further, we found that the seed-setting rates of 9311/*sss1-D* F_1_ and *sss1-D*/9311 F_1_ plants were ~54.8% and ~54.4%, respectively; and the plants of an F_2_ population (Q1, n = 160) derived from *sss1-D* and 9311 segregated plants of normal and low seed-setting rate in a 1:3 ratio (*x*^2^ = 0.075 < *x*^2^_0.05, 1_) (S1 Fig). These results indicated that the low seed-setting rate phenotype of *sss1-D* is caused by a single dominant mutation.

### Pollen tube growth is blocked in about half of the *sss1-D* pistils

To dissect the cellular defects for the low seed-setting rate phenotype of *sss1-D*, we compared the behavior of pollen tube growth after pollination. At 5 min after pollination (MAP), the germination rate of pollen grains and the ability of pollen tube generation in *sss1-D* were comparable to those of wild type (Fig 1H–1K and S2 Fig). However, reduced pollen tube growth was observed in *sss1-D* at 30 MAP and thereafter (Fig 1L–1O). At 120 MAP, about 87% wild type pistils had pollen tube tips arriving at the basal ovules and the micropyles, but only about 51% of the *sss1-D* pistils had pollen tube tips reaching the micropyles and about 49% of the *sss1-D* pistils had pollen tubes arrested in various positions in the styles (Fig 1P–1U). These results suggest that the low seed-setting rate of the mutant most likely results from blockage of pollen tube growth in the style. On the other hand, these results also suggest that once the growth barrier in the styles was overcome, the *sss1-D* pollen tubes could reach the micropyles. To confirm this, we analyzed a large number of embryo sacs at 24 h after pollination (HAP), when the double fertilization is completed in rice. In wild type, about 98% of the embryo sacs were normally fertilized, each with a multi-celled globular embryo and a layer of free endosperm nuclei. On the contrary, only about 46% of the embryo sacs were fertilized in *sss1-D* (S3 Fig). These observations are consistent with the reduced seed-setting rate of the *sss1*-*D* mutant (Fig 1B).

To further confirm this, we examined pollen tube growth in the pistils of hand-pollinated reciprocal crosses. When wild type was used as the pollen receiver, pollen tubes grew normally and ultimately reached the micropyles. However, when the *sss1-D* mutant stigmas were sprinkled with wild type or its own pollen grains, the same abnormalities were observed as those in the self-pollinated mutant pistils (Fig 1V). Taken together, our results demonstrated that the blocking of pollen tube growth in the styles and subsequent failure in double fertilization might be the main cause of low seed-setting rate in the *sss1-D* mutant.

To test the female transmission efficiency, we analyzed the genotype of each individual plant in another F_2_ population (Q2) derived from *sss1-D* and 9311 (n = 234). The result showed a 1:2:1 segregation ratio (*x*^2^ = 1.385 < *x*^2^_0.05, 2_) of homozygous wild type plants (59): heterozygous plants (124): homozygous *sss1-D* mutant plants (51), suggesting that both the wild type and mutant female gametophytes could be efficiently transmitted. These observations together suggest that the reduced seed-setting rate of the mutant is due to a defect in the female sporophytic tissue.

### *sss1-D* encodes OsCNGC13

**Fig 2:**
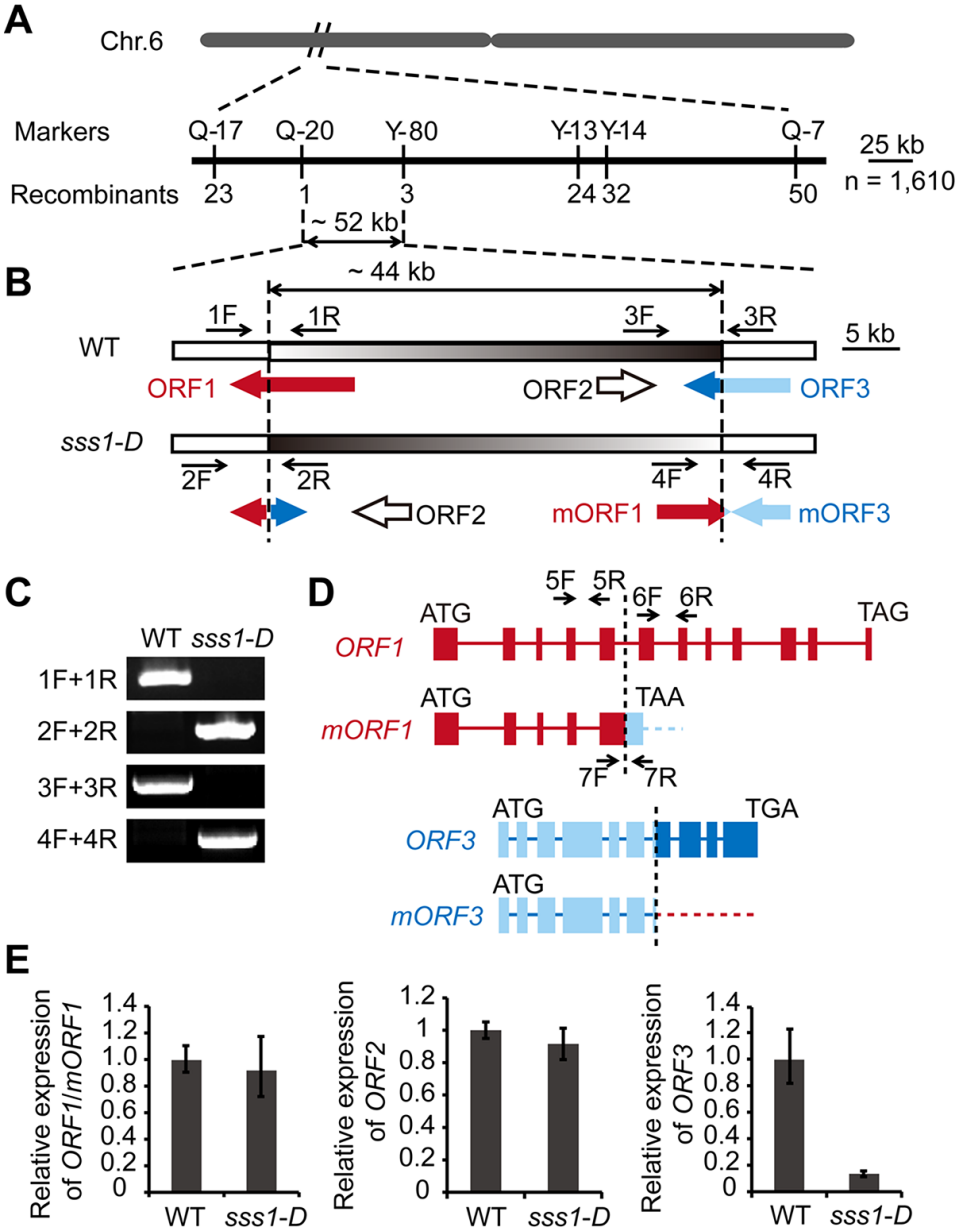
Molecular cloning. (A) The *sss1-D* locus was mapped to a ~52 kb region on the short arm of chromosome 6 between the markers Q-20 and Y-80. (B) Annotated open reading frames (ORF) in the ~52 kb region. Note that the inversion of a 44 kb genomic fragment in *sss1-D* disrupts both ORF1 and ORF3. (C) Verification of the inversion in *sss1-D*. Primers used for PCR analysis are shown in B. (D) A diagram of ORF1 and ORF3 in wild type and their mutant version in *sss1-D*. The inversion results in a truncated ORF1 with a premature stop (mORF1) and a stop codon-less ORF3 (mORF3). Filled box, exon; bold line, intron. (E) qRT-PCR analysis of the transcript levels of *ORF1*/*mORF1* (primer pair 5F/5R in D), *ORF2*, and *ORF3*. Data are means ± SD (n = 3).

A map-based cloning strategy was used to isolate the target gene locus using an F_2_ mapping population derived from the cross between *sss1-D* and the *indica* cultivar N22. The target gene locus was initially placed in an ~300-kb interval between the markers Q-17 and Q-7 on the short arm of rice chromosome 6 and was further restricted to a 52-kb genomic region flanked by the markers Q-20 and Y-80 (Fig 2A). Three putative open reading frames (ORF1-3) were predicted in this mapping region. *ORF1* (*LOC*_*Os06g10580*) is predicted to encode the rice Cyclic Nucleotide-Gated Channel 13 (OsCNGC13) with a pore-forming region and CNBD domain at the C-terminal region [15, 30]. *ORF2* (*LOC*_*Os06g10590*) is predicted to encode a putative uncharacterized expressed protein, and *ORF3* (*LOC*_*Os06g10600*) is predicted to encode a putative homeobox and START domain containing protein (http://www.tiger.org). Sequence analysis revealed that an ~44-kb genomic DNA segment was inverted and two ORFs (*ORF1* and *ORF3*) were interrupted in *sss1*-*D*, resulting in disruption of ORF3 and a mutated ORF1 (mORF1). ORF2 remained unchanged (Fig 2B and 2C). The inversion causes *mORF1* become a chimeric gene that is prematurely terminated (Fig 2D). Quantitative real-time reverse transcription (qRT)-PCR assay showed that the transcript levels of *ORF1*/*mORF1* and *ORF2* between wild type and *sss1-D* were comparable, while the expression of *ORF3* was dramatically reduced in *sss1-D* (Fig 2E).

**Fig 3:**
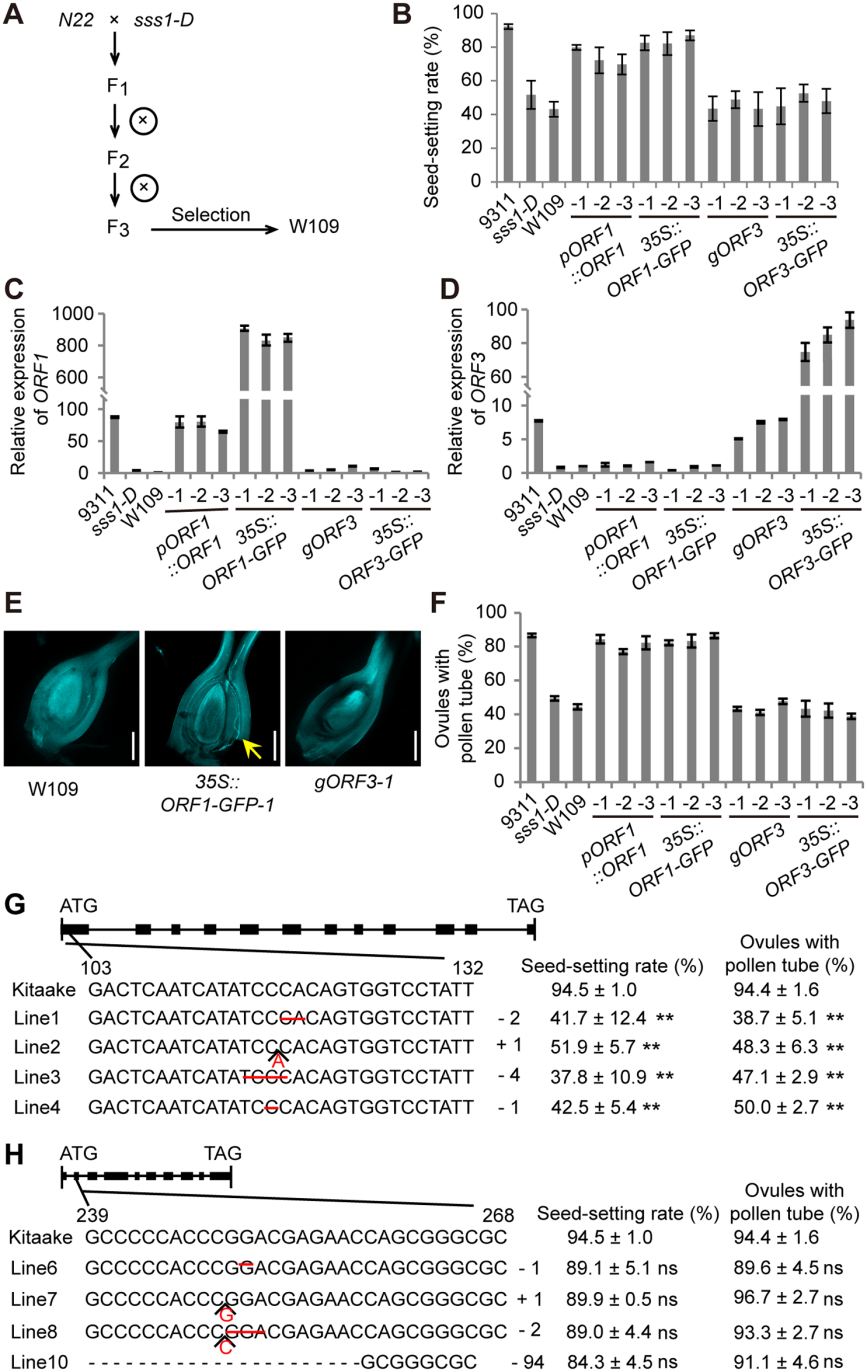
Functional complementation and gene knockout analyses. (A) Genetic scheme of W109 selection. (B-D) The seed-setting rate (B) and qRT-PCR analysis of *ORF1* (primer pair 6F/6R shown in Fig 2D) (C) and *ORF3* transcript levels (D) in W109 and the transgenic plants. Three independent transgenic lines individually transformed with *pORF1*::*ORF1*, *35S*::*ORF1-GFP*, *gORF3*, and *35S*::*ORF3-GFP* are shown. (E) Observation of pollen tube growth in W109, *35S*::*ORF1-GFP-1* transgenic plant, and *gORF3-1* transgenic plant. Arrow indicates the pollen tube tip. Scale bars, 300 μm. (F) Frequency of the pistils with at least one pollen tube reaching the ovaries at 120 MAP. (G and H) Sketch map of the mutations of *ORF1* (G) and *ORF3* (H) in knockout lines. The mutation site, the corresponding seed-setting rate, and the percentage of ovules with pollen tube of each line are shown. Minus (-) and plus (+) signs indicate the number of nucleotides deleted and inserted, respectively. Data are means ± SD (n > 5 in B; n = 3 in G and H); data in F are means ± SD from 3 replicates with > 30 pistils observed per replicate. ns, no significance; **P<0.01 by the Student’s *t* test.

To test whether the alterations in *ORF1* or *ORF3* might be responsible for the mutant phenotype, we performed a genetic complementation assay. As the *sss1-D* mutant (in an *indica* background) was difficult for transformation, we individually transformed *pORF1*::*ORF1*, *35S*::*ORF1-GFP*, *gORF3*, and *35S*::*ORF3-GFP* into W109, a rice line derived from the N22/*sss1-D* F_3_ population and is homozygous at the *sss1-D* locus in the N22 genetic background (Fig 3A). The low seed-setting rate of W109 was largely rescued when *ORF1* was expressed (either driven by its endogenous promoter or by the *CaMV 35S* promoter); while the expression of *ORF3* (driven by its endogenous promoter or the *CaMV 35S* promoter) showed no effect (Fig 3B–3F). In addition, knockout of *ORF1* by CRISPR/Cas9 in the wild type Kitaake caused much reduced seed-setting rates (ranging from 37.8 ± 10.9% to 51.9 ± 5.7%) and much decreased percentages of the ovules with pollen tube (ranging from 38.7 ± 5.1% to 50.0 ± 2.7%) in all positive transgenic lines, while knockout of *ORF3* showed no effect on the seed-setting rate (Fig 3G and 3H). These results suggest that *ORF1*/*OsCNGC13* represents the target gene.

### The dominant nature of *sss1-D* is likely caused by haploinsufficiency

**Fig 4:**
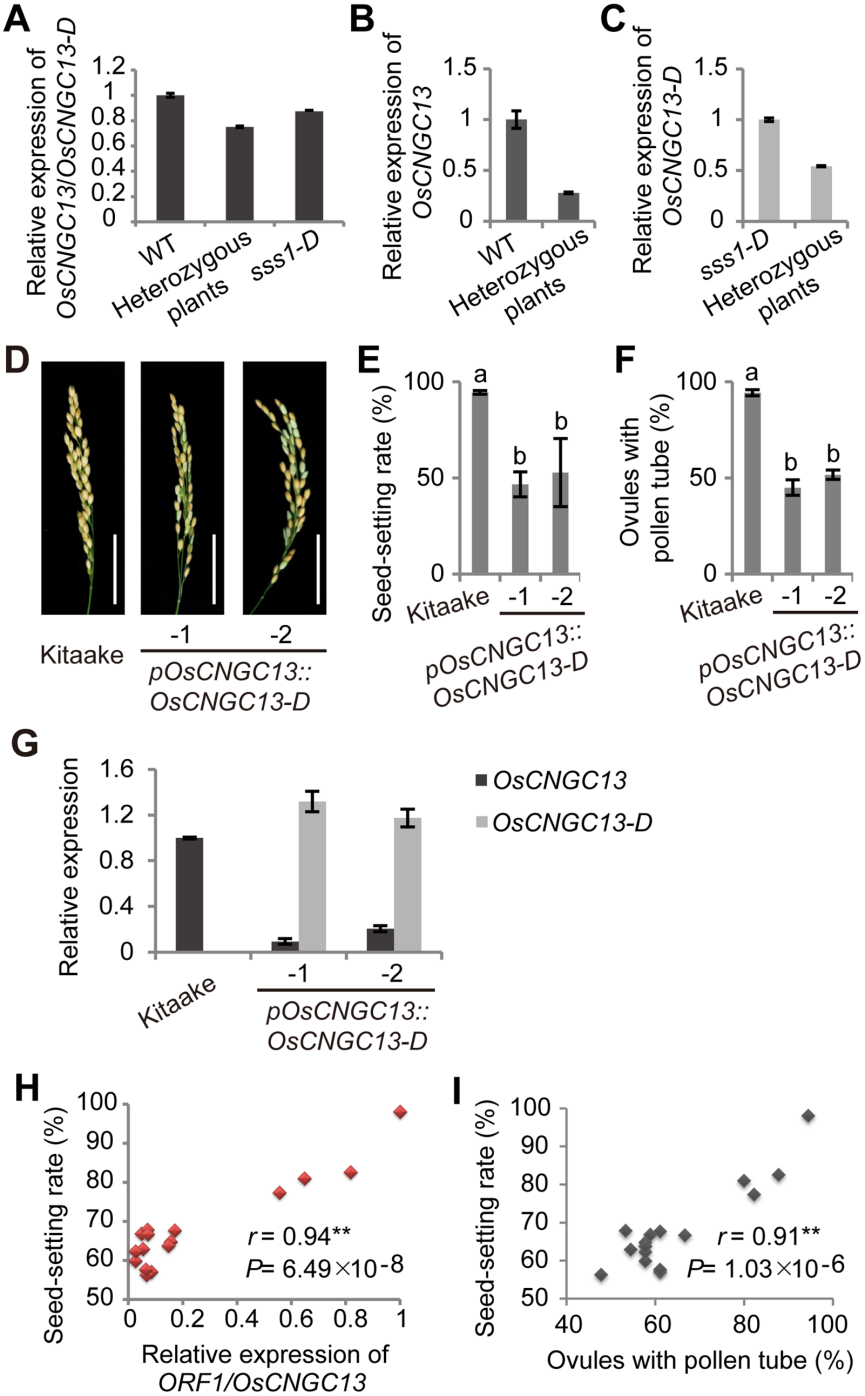
The expression of *OsCNGC13* (*ORF1*) positively affects the plant seed-setting rate. (A) qRT-PCR analysis of *OsCNGC13* and *OsCNGC13-D* in WT, *sss1-D*, and heterozygous plants with the 5F/5R primer pair shown in Fig 2D. Note that the expression of *OsCNGC13* and *OsCNGC13-D* in the heterozygous plants was significantly reduced. (B and C) qRT-PCR analysis of *OsCNGC13* and *OsCNGC13-D* in WT, *sss1-D* and heterozygous plants with allele-specific primers. Unequal reduction of expression of *OsCNGC13* and *OsCNGC13-D* is observed in the heterozygous plant as normalized to WT (B) and *sss1-D* (C). (D) Comparison of mature panicles of wild type Kitaake and two independent lines (-1 and -2) of *pOsCNGC13*::*OsCNGC13-D*. Scale bars, 5 cm. (E and F) The seed-setting rate (E) and the percentages of ovules with pollen tube (F) in Kitaake and the transgenic plants. Data in E are means ± SD (n > 5). Data in F are means ± SD from 3 replicates with > 30 pistils observed per replicate. Different letters indicate a significant difference at *P* < 0.01 by the Student’s *t*-test. (G) qRT-PCR analysis of *OsCNGC13* and *OsCNGC13-D* in Kitaake and the transgenic plants. The primer pairs used in A (5F/5R for both *OsCNGC13* and *OsCNGC13-D*), B (6F/6R specific for *OsCNGC13*), C (7F/7R specific for *OsCNGC13-D*), G (6F/6R and 7F/7R) are shown in Fig 2D. Data are means ± SD (n = 3). (H) Positive correlation between the seed-setting rates and expression levels of *OsCNGC13* in several RNAi transgenic lines. (I) Positive correlation between the seed-setting rates and the percentages of ovules with pollen tube in several RNAi transgenic lines. *r* value is based on two-tailed Pearson correlation analyses.

Since the low seed-setting rate phenotype of *sss1-D* mutant was dominant, we reasoned that it might be caused by a dominant-negative effect of the mORF1 protein. Compared with the normal OsCNGC13, *mORF1* is predicted to encode a product (OsCNGC13-D) with only 440 amino acids with five transmembrane domains at the N-terminus. The C-terminal pore-forming region and CNBD domain were lost due to premature termination (S4A and S4B Fig). To test whether the mutated OsCNGC13-D protein may interfere with the normal function of OsCNGC13, we first examined whether OsCNGC13 can form homodimer and whether OsCNGC13-D can interact with OsCNGC13. Both yeast two-hybrid assay and bimolecular fluorescence complementation (BiFC) assay failed to detect homomeric or heteromeric interaction between OsCNGC13-D and OsCNGC13 (S5 and S6 Figs), indicating that it is unlikely OsCNGC13-D negatively affects OsCNGC13 protein function directly. Next, we compared the *OsCNGC13* transcript levels in the wild type 9311, 9311/*sss1-D* F_1_ plants (heterozygous plants) and *sss1-D* homozygous mutant plants. The result showed that, compared with the wild type or the homozygous mutant, the transcript level of *OsCNGC13* in the heterozygous plants was reduced (Fig 4A). qRT-PCR analysis using allele-specific primer pair also showed that expression of the wild type *OsCNGC13* allele, but not the mutant *OsCNGC13-D* allele, was dramatically reduced in the heterozygous plants (Fig 4B and 4C). Moreover, we artificially expressed *OsCNGC13-D* in wild type Kitaake. Positive transgenic plants showed reduced seed-setting rates and decreased percentages of ovules with pollen tube (Fig 4D–4F), which is similar to the mutant phenotypes of *sss1-D*. More importantly, we found that the expression of *OsCNGC13* allele was significantly reduced in the transgenic lines (Fig 4G). Furthermore, we observed a correlation between the expression levels of *OsCNGC13*, percentages of ovules with pollen tube, and the reduced seed-setting rates in the *OsCNGC13* RNAi lines (Fig 4H and 4I and S2 Table). These observations together suggest that the dominant nature of *sss1-D* mutation might be caused by haploinsufficency resulting from the suppression of *OsCNGC13* by the mutated *OsCNGC13-D* allele.

### Expression pattern of *OsCNGC13* and subcellular localization of its product

**Fig 5:**
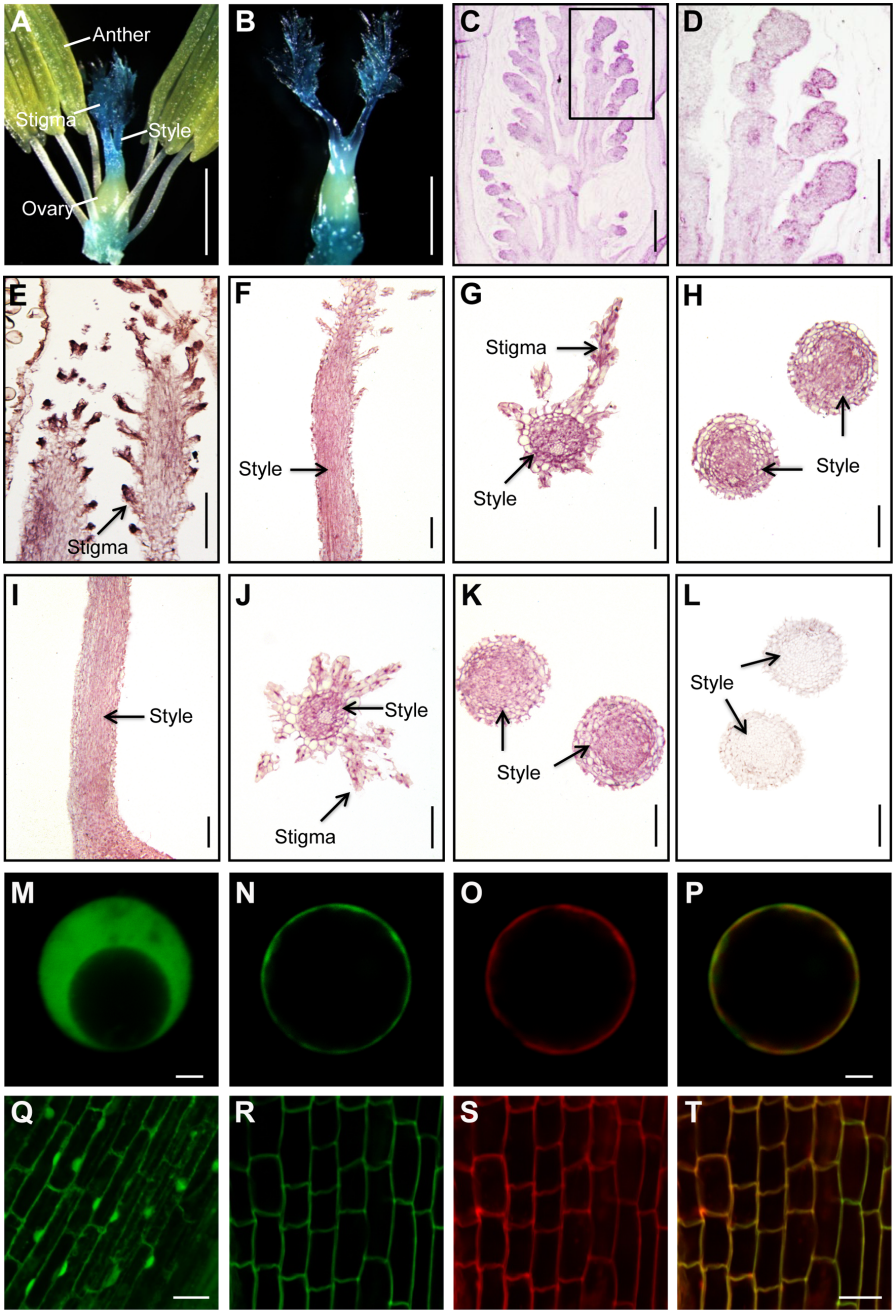
*OsCNGC13* mRNA expression and subcellular localization of its protein. (A and B) GUS staining of the spikelets after removing the lemma and palea. Mature pistil before flowering (A) and pistil at 30 MAP (B) are shown. (C-L) *In situ* hybridization analysis. Longitudinal sections of the floral primodia (C and D), stigma (E) and style (F) before flowering, and the style of the pistil at 30 MAP (I) are shown. (D) The magnified image of the selected area in C. Transverse sections of the stigmas (G and J) and styles (H, K and L) of the pistils before flowering (G and H) and the pistils at 30 MAP (J-L) are shown. (L) Negative control with sense probe. (M-P) Localization of GFP protein (M), OsCNGC13-GFP fusion protein (N), and PIP2;1-mCherry fusion protein (O) in rice protoplasts. (P) The merged image of N and O. (Q-T) Localization of GFP protein in the root tip cells of transgenic rice plants expressing 35S promoter-driven *GFP* (Q) and localization of OsCNGC13-GFP fusion protein in the root cells of the transgenic rice plants expressing *35S*::*OsCNGC13-GFP* (R). (S) Cellular outlines of the root cells were stained with FM4-64 for 5 min on ice. (T) The merged image of R and S. Scale bars, 1 mm in (A and B); 200 μm in (C and D); 50 μm in (E-L); 10 μm in (M-P); 20 μm in (Q-T).

Search of the microarray expression data from the RiceXpro database (http://ricexpro.dna.affrc.go.jp/) revealed that *OsCNGC13* is expressed in various tissues with peak expression in the pistils (S7A Fig). To examine its expression in more detail, we generated pOsCNGC13-GUS reporter gene transgenic plants. Histochemical staining detected expression in the vascular tissues of the primary root, leaf, leaf sheath, spikelet hulls (S7B–S7H Fig), the stigmas and styles of the mature pistils, and the pistils at 30 MAP (Fig 5A and 5B). Further, *in situ* hybridization confirmed preferential expression of *OsCNGC13* in the floral primodia, the stigmas and styles of mature pistils before and after pollination (Fig 5C–5L). To determine the subcellular localization of the OsCNGC13 protein, we transiently expressed the OsCNGC13-GFP fusion protein in rice protoplasts. The result showed that the control GFP signal was dispersed in the cytosol, while the green fluorescence of OsCNGC13-GFP merged well with red signal of the plasma membrane marker, PIP2;1-mCherry [31], indicating that OsCNGC13 was located to the plasma membrane (Fig 5M–5P). Similar result was also found in the tobacco leaf epidermal cells and mesophyll protoplasts transiently expressing the *OsCNGC13-GFP* construct (S8 Fig). Consistent with these observations, the OsCNGC13-GFP fusion protein was also detected in the cell perimeter in the *35S*::*OsCNGC13-GFP* transgenic plants (Fig 5Q–5T). Together, these results indicate that OsCNGC13 is a plasma membrane localized protein.

### OsCNGC13 has permeability to inward Ca^2+^ and is indispensable for the [Ca^2+^]_cyt_ accumulation in the style

**Fig 6:**
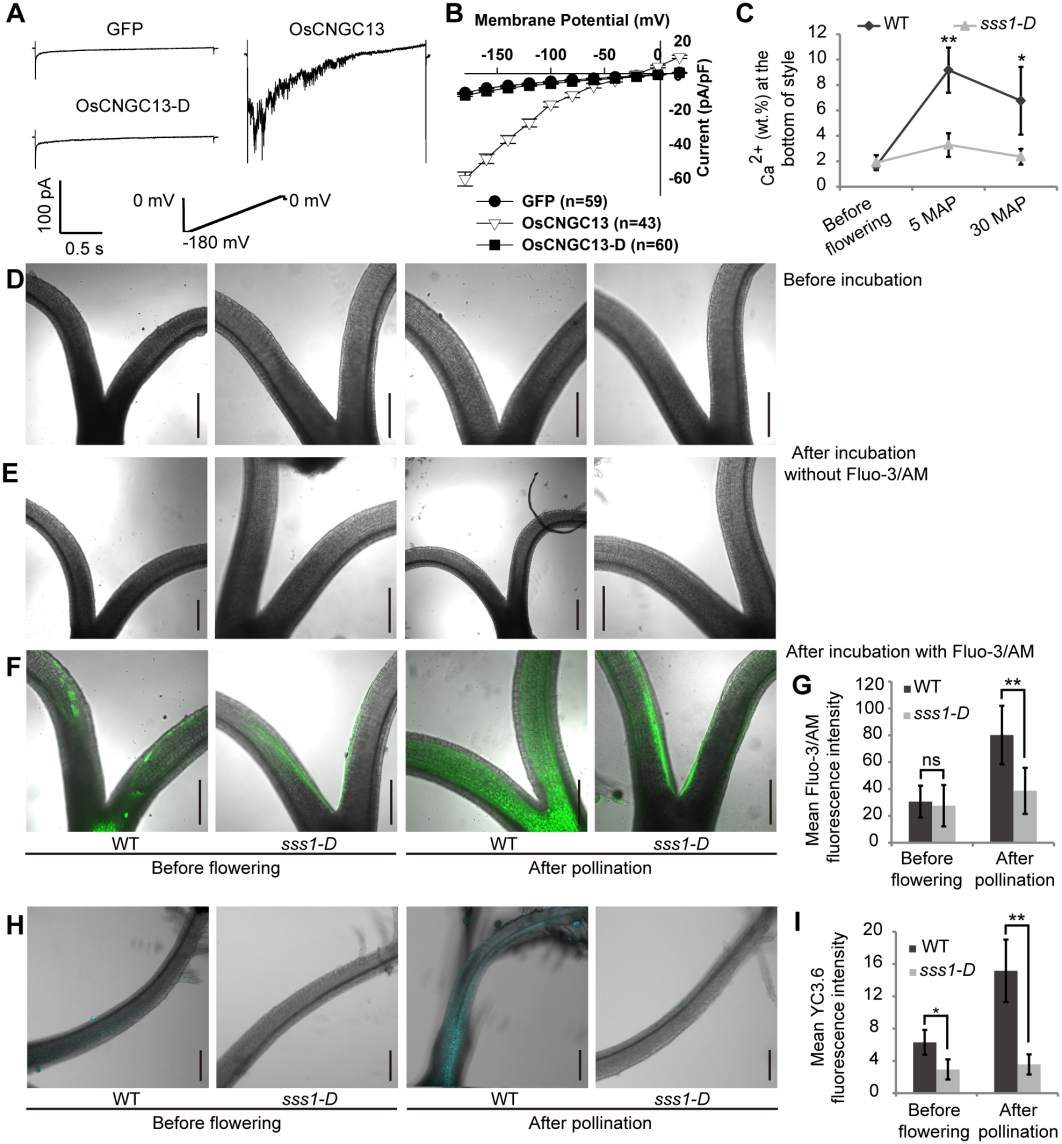
OsCNGC13 exhibits permeability to inward Ca^2+^ in HEK293 cells and is required for [Ca^2+^]_cyt_ accumulation in the style. (A) Patch-clamp whole-cell recordings of inward Ca^2+^ currents in HEK293 cells transfected with *GFP* (as a control), *OsCNGC13*, or *OsCNGC13-D*. The voltage protocols, as well as time and current scale bars for the recordings are shown. (B) The I-V relationship of the steady-state whole-cell inward Ca^2+^ currents. The data are derived from the recordings shown in A, and presented as means ± SD. (C) Style Ca^2+^ content measurement using SEM-EDX. Increased Ca^2+^ concentrations are detected in WT but not in *sss1-D* after pollination. (D-G) Fluo-3/AM showing Ca^2+^ accumulation in the styles. Images of styles before Fluo-3/AM incubation (D), after incubation in the buffer without Fluo-3/AM (E), and after Fluo-3/AM incubation (F) are shown. Note that the samples before incubation and the samples incubated in the buffer without Fluo-3/AM were performed as the negative controls, both of which showed no fluorescence. (G) Quantification of Fluo-3/AM fluorescence intensity. (H and I) Ca^2+^ accumulation in the styles indicated by the YC3.6 protein fluorescence. (I) Quantification of YC3.6 fluorescence intensity. Data are means ± SD (n = 4 in C; n = 9 in G; n = 6 in I). *P<0.05 and **P<0.01 by the Student’s *t* test. Scale bars, 100 μm.

Multiple *Arabidopsis* CNGCs have been demonstrated to mediate the Ca^2+^ currents virtually [18, 28, 32, 33]. To determine whether OsCNGC13 had permeability to Ca^2+^, we conducted patch-clamp whole-cell recording in HEK293 cells to measure the OsCNGC13-mediated Ca^2+^ currents. Remarkable whole-cell inward Ca^2+^ currents were detected when OsCNGC13, but not the negative control GFP or the OsCNGC13-D protein, was transfected into HEK293 cells (Fig 6A and 6B). On the other hand, OsCNGC13 had barely detectable permeability to K^+^ compared with the positive control OsAKT1 [34] (S9 Fig).

Previous studies reported that application of compatible pollens to the stigma papilla cells could trigger an increase in cytosolic free calcium [35, 36]. Thus, we speculated whether a similar response might happen in the style when pollinated. Scanning electron microscopy-energy dispersive x-ray spectrometry (SEM-EDX) of the cross-sections of the styles revealed that *sss1-D* mutant had continuous low Ca^2+^ concentrations in the style, whereas the wild type showed a maximum net value of ~9.2% at 5 MAP at the bottom of the styles, and this high Ca^2+^ concentration continued till 30 MAP (Fig 6C). Staining with Fluo-3 acetoxymethyl (AM) ester, a Ca^2+^-sensitive fluorescent dye [37], showed that in the styles of mature pistils before flowering, the [Ca^2+^]_cyt_ maintained at a relative low level in both the wild type and the *sss1-D* mutant (Fig 6D–6G). After pollination, the fluorescence intensity in the wild type styles increased gradually, while in the mutant, only background signal could be detected and the fluorescence intensity remained at a low level (Fig 6F and 6G). Similar results were observed when yellow cameleon 3.6 (YC3.6) was used as a ratiometric fluorescent calcium indicator [38] and when potassium pyroantimonate was used to precipitate the intracellular calcium [39] (Fig 6H and 6I and S10 Fig), respectively. These results together demonstrate that the OsCNGC13 is indispensable for triggering [Ca^2+^]_cyt_ accumulation in the style after pollination.

### The *sss1*-*D* mutant has altered ECM components

**Fig 7:**
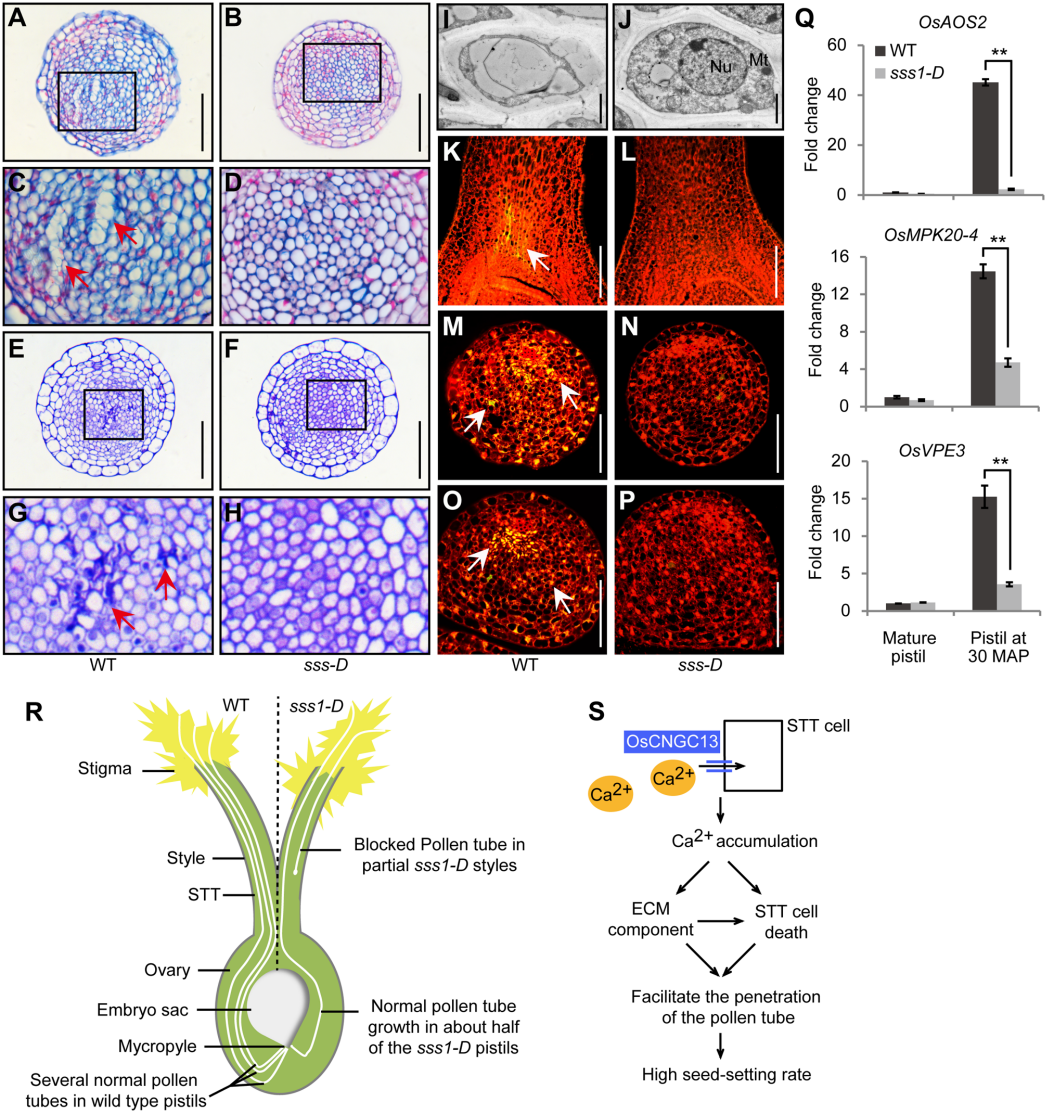
Defective PCD in styles of *sss1-D* during pollen tube growth and a proposed model of OsCNGC13 function. (A-H) Transverse paraffin (A-D) and plastic (E-H) sections at the middle of the style. The boxed regions in A, B, E, and F are magnified in C, D, G, and H, respectively. Intercellular space (red arrows), as a result of PCD, is observed in WT but not in *sss1-D*. (I and J) TEM images of cells at the bottom of the style, showing a collapsed cell in WT (I) but not in *sss1-D* (J). Nu, nucleus; Mt, mitochondria. (K-P) TUNEL assay shows that DNA fragmentation signal (white arrow) is visible in WT but not in *sss1-D*. Longitudinal sections of the bottom (K and L), transverse sections at the middle (M and N) and bottom parts (O and P) of the style are shown. (Q) qRT-PCR analysis of the transcript levels of the genes related to PCD. Pollination-triggered expression of these genes is significantly reduced in *sss1-D*. All samples used in A-P were collected at 30 MAP. Data in Q are the mean ± SD (n = 3). **P < 0.01 by the Student’s *t*-test. Scale bars, 50 μm in (A, B, E, F and K-P); 1 μm in (I and J). (R) A sketch illustrating the patterns of pollen tube growth in the pistils of the wild type and the *sss1-D* mutant. The growth of pollen tube is normal in almost all the wild type pistils, while the pollen tube is blocked in about half of the *sss1-D* pistils. (S) A proposed model of OsCNGC13 function. OsCNGC13 is localized on the plasma membrane of the STT cells and plays an important role in linking [Ca^2+^]_cyt_ accumulation in the style after pollination, ECM components modification and PCD in the style to facilitate the penetration of the pollen tube, successful double fertilization, and consequently high seed-setting rate.

In angiosperms, specialized cell files in the style, which form the STT, define the paths of pollen tubes to approach the ovary and produce a complex mixture of polysaccharides, glycoproteins, and glycolipids that is known as the ECM. The ECM has been reported to provide guidance signals (i.e., Ca^2+^ flux capacitor) as well as nutrients for pollen tube growth [40–42]. Staining with H33342, a nucleic acid fluorescent dye [43], showed no difference in the overall cellular structure of the style tissue between the wild type and the mutant (S11A–S11D Fig). Alcian blue, a stain for acidic polyanions [11], was used to indicate the acidic polysaccharides (major components of the ECM). The result showed that after pollination, most tissues and cells (except for the vascular tissues and epidermal cells) in the sections of wild type could be stained deeply, while the staining was weaker in the corresponding regions of the mutant styles (Fig 7A–7D). Moreover, weak staining was also observed in the mature pistils before flowering (S11E–11L Fig). These results demonstrate that in *sss1*-*D*, the female reproductive tract is defective in ECM components.

### PCD is delayed in the pollinated styles of the *sss1*-*D* mutant

Previous studies had also shown that pollination triggered calcium signaling could lead to modification of ECM components and the death and degeneration of transmission tract cells, allowing the pollen tube to penetrate the style and reach the ovules for fertilization [10, 11, 44, 45]. Both plastic section observation and the terminal deoxynucleotidyl transferase-mediated dUTP nick-end labeling (TUNEL) assay showed no cell degradation in the styles of wild type or *sss1-D* before flowering (S11M–11T Fig). Cytological examination revealed that at 30 MAP, the wild type style had intense staining and notable intercellular space, indicating that some style cells had broken down after pollination, while in the *sss1*-*D* mutant, all the cells remained intact (Fig 7A–7H). To confirm this, we performed transmission electron microscopy (TEM) analysis of the transverse sections of the wild type and mutant styles. Some bottom style cells in the wild type pistil lacked contents inside, whereas all the cells had well defined organelles and abundant cytosolic contents in the mutant style (Fig 7I and 7J). Consistent with the above results, the TUNEL assay showed that positive signals were detected in the wild type style, but not in the mutant style (Fig 7K–7P). Further, qRT-PCR analysis revealed that the expression of several genes related to PCD [46–48] was elevated in the wild type pistils at 30 MAP, but not in the *sss1-D* mutant pistils (Fig 7Q). All these results indicate that STT cell death is defective in the *sss1*-*D* mutant after pollination.

## Discussion

In this study, we showed that *sss1-D* represents a novel dominant low seed-setting rate mutant in rice. Our cytological studies suggest that the low seed-setting rate of *sss1-D* is caused by blockage of pollen tube growth in about half of the pistils (Fig 1H–1V). Further, our reciprocal cross assays, together with transmission analysis, pollen fertility assay, and cytological observations, demonstrate that the blocking of pollen tube growth in *sss1-D* styles is caused by a maternal defect. Similar cases have been reported before. *PTB1*, which encodes a RING type E3 ubiquitin ligase in rice, positively regulates panicle seed-setting rate and grain yield by promoting pollen tube growth in the STT [7]. Differently, *ptb1* exhibits extreme sterility because all the pollen tubes are blocked in the STT. While the seed-setting rate of the *sss1-D* mutant is ~51%, and pollen tube is blocked in ~51% of the mutant pistils at 120 MAP.

Our molecular cloning and functional complementation assay revealed that a genomic inversion in *OsCNGC13* forming *OsCNGC13-D* is responsible for the mutant phenotype. We show that OsCNGC13 possesses all the classical domains of the CNGC family, and is localized to the plasma membrane, similar to AtCNGC2, AtCNGC12, AtCNGC14, AtCNGC17 and AtCNGC18 [49]. However, it should be noted that some CNGCs have been localized to other subcellular localizations. For example, three *Medicago truncatula* CNGC proteins (MtCNGC15a/b/c) were found to be localized to the nuclear membrane [33]. Surprisingly, AtCNGC19 and AtCNGC20, the closest homologs of OsCNGC13 in Arabidopsis [30] (S12 Fig), were shown to be localized in vacuole membrane in one study [50], while AtCNGC20 was localized to the plasma membrane in another study [51]. The distinct subcellular localizations of CNGC proteins might be consistent with their broad range of biological functions.

In general, CNGC have been shown to form tetrameric channels in animals [52]. Similarly, AtCNGC11/12, AtCNGC2 and AtCNGC4 have been shown likely to form homotetramer and heterotetramer as well [53, 54]. Recently, AtCNGC17 was shown to form homodimer or oligomer and can interact with AHA1, AHA2, and BAK1 to regulate Arabidopsis growth, suggesting that AtCNGC17 functions in a protein complex (es) [55]. Somewhat unexpectedly, our Y2H (S5 Fig) and BiFC (S6 Fig) experiments failed to detect direct homomeric or heteromeric interaction between OsCNGC13-D and OsCNGC13, suggesting that OsCNGC13-D is unlikely to affect the function of OsCNGC13 directly. However, whether OsCNGC13 can form heteromultimer with other members of the rice CNGC family *in vivo* remains to be investigated in future studies.

Notably, we found that the mutated allele, *OsCNGC13-D*, causes a significant reduction in the expression level of the wild type *OsCNGC13* allele in the heterozygous background and in wild type plants expressing *OsCNGC13-D* driven by its native promoter (Fig 4A–4G). Therefore, we deduced that the dominant nature of *sss1-D* mutant is likely caused by haploinsufficiency [56, 57]. The precise mechanism of this regulation is currently not clear. It is possible the *OsCNGC13-D* allele might become a paramutagenic allele to alter the expression of the other allele at the same genetic locus in a heritable manner [58]. Further studies are required to resolve this issue.

We also show that OsCNGC13 can mediate inward Ca^2+^ current (Fig 6A and 6B). Consistent with being a maternal sporophytic factor required for normal pollen tube elongation in the styles, we found that *OsCNGC13* is preferentially expressed in the stigmas and styles (Fig 5A–5L). The *sss1-D* mutant is defective in triggering [Ca^2+^]_cyt_ accumulation in the style after pollination (Fig 6C–6I and S10 Fig). These results suggest that OsCNGC13 is likely required for Ca^2+^ influx in STT cells. Interestingly, we also observed altered ECM components and delayed STT cell death in the *sss1-D* mutant pistils (Fig 7A–7Q). These observations are consistent with the earlier findings that characteristic calcium signaling in the principal sporophytic cells is essential for successful double fertilization [59, 60] and that pollination triggered calcium signaling is functionally linked with ECM production/modification and PCD of STT cells to facilitate pollen tube growth in the stylar tissues [40, 41, 61]. In support of this notion, several AtCNGCs have been shown to be involved in the process of PCD. For example, AtCNGC2 was shown to possess a Ca^2+^ influx channel activity that can mediate Ca^2+^ influx into leaf cells [19, 22]. The leaves of null mutant of *AtCNGC2*, *defense*, *no death* (*dnd1*), display delayed PCD when challenged with pathogens [22]. Null mutants of *AtCNGC4* (the closest paralog of *AtCNGC2*) exhibit remarkably similar phenotypes to *dnd1* [62, 63], supporting a notion that AtCNGC2 and AtCNGC4 may form a heteromeric Ca^2+^ channel complex [54]. Similarly, several recent studies also documented evidence for a role of AtCNGC11 and AtCNGC12 in PCD and plant immunity [21, 23, 24, 26]. Thus, we speculated that the delayed cell death in the style caused by the OsCNGC13 defect might ultimately explain the blockage of pollen tube growth in the *sss1-D* mutant. Similar observations have been made with the Arabidopsis *ntt* (*no transmitting tract*) mutant, which has reduced fertility due to defects in ECM production and formation of the ovary transmitting tract, and consequently blockage of pollen tube growth [11]. Although the mechanistic details need to be further elucidated, our results suggest that OsCNGC13 play an important role in linking [Ca^2+^]_cyt_ accumulation, ECM components modification, and PCD in the style after pollination to allow proper pollen tube growth and successful double fertilization (Fig 7R and 7S), which ultimately affects seed-setting rate in rice.

## Materials and methods

### Plant materials and growth conditions

The *sss1*-*D* mutant was identified from a ^60^Co-irradiated M_2_ population of the *indica* rice (*Oryza sativa* L.) cultivar 9311. The *sss1*-*D* mutant was crossed with 9311 to produce two F_2_ populations (Q1 and Q2) in 2012 and 2013, respectively, which were adopted for genetic and gametophyte transmission analyses. An F_2_ mapping population was generated from a cross between the *sss1*-*D* mutant and the *indica* cultivar N22. All plants were grown in paddy fields during the normal growing seasons or in a greenhouse at the Chinese Academy of Agricultural Sciences, in Beijing.

### Examination of pollen grain germination on the stigma

More than 30 pollinated pistils of the mutant and wild type were collected and fixed in FAA (containing an 18:1:1 (by vol.) mixture of 70% ethanol, formalin and acetic acid) for 24 h. The spikelets were then processed through an ethanol series (70, 50, and 30%) and washed three times with distilled water. The spikelets were incubated in 10 mol L^-1^ sodium hydroxide for 8 min at 56°C and then washed with distilled water three times and stained in 0.1% aniline blue solution for 12 h. Finally, the samples were examined using a scanning confocal microscope (ZEISS LSM 700).

### Embryo sac observation

Embryo sac development analysis was performed as described previously [43].

### Map-based cloning

To map the target gene locus, 1610 mutant individuals were collected from the F_2_ population of *sss1*-*D* and N22. The selected mutant plants and newly developed molecular markers were used for primary and fine mapping. Inversion in the mutant was confirmed using PCR with the primer pairs 1F/1R, 2F/2R, 3F/3R, and 4F/4R. All primer sequences used for the map-based cloning are listed in S3 Table.

### qRT- PCR and 3’ RACE

Total RNA was extracted from fresh samples using a plant RNA extraction kit (Tiangen Co., Beijing, China) according to the manufacturer’s instructions. 1 μg of total RNA of each sample was reverse transcribed to cDNA using a reverse transcription kit (SuperScript II; TaKaRa). qRT-PCR was performed using a SYBR Premix Ex TaqTM kit (TaKaRa) on an ABI prism 7500 Real-Time PCR System. The rice *ubiquitin* gene (*Os03g0234200*) was used as a reference gene with the primer pair Ubq. Each RNA sample was extracted from a pool of tissues collected from at least three individual plants.

The 3’ RACE was performed using the SMARTer RACE cDNA amplification kit (Clontech Laboratories), according to the manufacturer’s instructions. The first-strand cDNA was synthesized from total RNA of 7-d-old *sss1-D* mutant seedlings. The sequence of the obtained PCR fragment was used as a template for direct sequencing. All primer sequences used here are listed in S3 Table.

### Vector construction and rice transformation

For genetic complementation, a 2.9-kb genomic fragment upstream of the ATG start codon of *ORF1* was amplified by PCR using the wild type genomic DNA as the template, and then the full-length CDS of *ORF1* was amplified from the wild type cDNA. Both fragments were then cloned into the pCAMBIA1305 vector to generate the *pORF1*::*ORF1* construct. A 6.7-kb *ORF3* genomic fragment spanning the entire coding region, 2000-bp upstream sequence, and 500-bp downstream sequence was amplified and recombined into the pCAMBIA1305 vector to generate the *gORF3* construct. The full-length CDSs of *ORF1* and *ORF3* were cloned into the pCAMBIA1305-GFP vector (generated by insertion of a *Sac*I-*Sal*I fragment containing the GFP expression cassette of pAN580 into the pCAMBIA1305 vector) to generate the *35S*::*ORF1-GFP* and *35S*::*ORF3-GFP* constructs, respectively. Subsequently, the plasmids *pORF1*::*ORF1*, *gORF3*, *35S*::*ORF1-GFP*, and *35S*::*ORF3-GFP* were individually introduced into the *Agrobacterium tumefaciens* strain EHA105 and used to infect the calli of W109. Transgenic roots used for subcellular localization were analyzed by scanning confocal microscope (ZEISS LSM 700).

For knockout lines, one 20-bp gene-specific sequence for *ORF1* and *ORF3* was synthesized and cloned into the entry vector pOs-sgRNA, and then cloned into the gateway destination vector pOs-Cas9, respectively. The resulting plasmids were individually introduced into Kitaake. Positive lines were confirmed by PCR followed by sequencing.

For expression of *mORF1* in wild type, a 2.9-kb promoter fragment of *ORF1* was amplified from the wild type, and then 1.3-kb full-length *mORF1* was amplified from the *sss1*-*D* cDNA. Both fragments were cloned into the pCAMBIA1305 vector to generate the *pORF1*::*mORF1* construct, which was introduced into Kitaake by Agrobacterium-mediated transformation.

The construct pCUbi1390-^Δ^FAD2 (ubiquitin promoter and a FAD2 intron inserted into pCAMBIA1390) was used as the RNAi vector [64]. Both sense and anti-sense versions of a specific 500-bp fragment from the coding region of the *ORF1* were amplified and successively inserted into pCUbi1390-^Δ^FAD2, to form the RNAi construct *pUbi-RNAiORF1*, which was introduced into Kitaake.

For expression pattern analysis, the 2.9-kb *OsCNGC13* promoter fragment was amplified by PCR and cloned into the binary vector pCAMBIA1305 to generate the *pOsCNGC13-GUS* construct. The construct was introduced into Kitaake. Samples of transgenic lines were observed with a stereo microscope (Leica MZ16), and photographed (Leica DFC490) after GUS histochemical staining. The primers and sequences used for constructing these vectors are listed in S3 Table.

### Sequence alignment

Gene prediction was performed using the Rice Genome Automated Annotation System (http://ricegaas.dna.affrc.go.jp/). Sequence analysis of OsCNGC13 and OsCNGC13-D was performed using the SOSUI program (http://bp.nuap.nagoyau.ac.jp/sosui/). Amino acid sequences of OsCNGC13 and OsCNGC13-D were used for alignment using the ClustalX 2.01 program with default settings. The BioEdit software was also used to perform multiple sequence alignments to confirm the ClustalX data output.

### Protein–protein interaction analysis

The DUALhunter system (Dualsystems Biotech) was used for yeast two-hybrid assay. The coding sequences of *OsCNGC13*, *OsCNGC13*-*D* and *OsCNGC12* were amplified and fused to the Cub-LexA-VP16 fragment in the pDHB1 and pXGY18 vector and the Nub fragment in the pPR3-N and pXGY17 vector, respectively. Test constructs were transformed into the yeast strain NMY51 according to the manufacturer’s instructions. The growth state of each transformant was examined on the QDO (SD/-Trp/-Leu/-His/-Ade) medium.

For the BiFC assay, the coding sequences of *OsCNGC13*, *OsCNGC13*-*D* and *OsCNGC12* were cloned into the binary BiFC vectors pSPYNE173 and pSPYCE(M), respectively. *AtCNGC2* was co-expressed with *AtCNGC4* as the positive control [54]. For transient expression, the *Agrobacterium tumefaciens* strain EHA105 carrying the BiFC constructs were co-infiltrated into *N*. *benthamiana* leaves with the p19 strain and the PM marker, PIP2;1-mCherry fusion protein [31]. Infiltrated leaves were observed 48–72 h after infiltration using a laser scanning confocal microscope (ZEISS LSM 700). The eYFP and mCherry fluorescent signals from the expressed fusion constructs were monitored sequentially. The excitation and detection wavelengths for eYFP and mCherry are 514 and 587 nm for excitation, and 527 and 610 nm for detection, respectively. All primer sequences used for plasmid construction are listed in S3 Table.

### RNA *in situ* hybridization

Young inflorescences at 2 weeks before flowering and spikelets at various stages were fixed using RNase-free FAA for 12 h at 4°C, dehydrated through an ethanol series, and then embedded in paraffin (Paraplast Plus, Sigma). A 252-bp *OsCNGC13* region was amplified and subcloned into the pGEM–T Easy vector (Promega), which was used as a template to generate both the antisense and sense RNA probes. DIG Northern starter kit (Roche) was used to prepare the digoxigenin-labeled RNA probes. Slides were observed under light microscopy (Leica DM5000B), and photographed using a Micro Color charge-coupled device camera (Apogee Instruments). All primer sequences used here are listed in S3 Table.

### Subcellular localization of OsCNGC13 protein

The full-length CDS of *ORF1* was amplified from the wild-type cDNA and cloned into the pA7-GFP vector to form translational fusion with the N-terminus of the green fluorescent protein (GFP). As a positive marker, the cDNA of a previously characterized plasma membrane protein, PIP2;1 [31], was fused to the mCherry gene to generate *35S*::*PIP2;1*-*mCherry*. Both constructs were co-transformed into rice protoplasts and incubated in the dark at 28°C for 16 h before examination. Meanwhile, the binary vectors *35S*::*ORF1-GFP* and pCAMBIA1305-GFP were introduced into *N*. *benthamiana* leaves, respectively. Confocal imaging analysis was performed using a laser scanning confocal microscope (ZEISS LSM 700). All primer sequences used here are listed in S3 Table.

### Cell culture, transfection, and whole-cell recording

The patch-clamping recordings from HEK293 cells were performed as described previously [34] HEK293 cells (ATCC) were cultured in DMEM (Dulbecco’s modified eagle medium) with 4500 mg L21 glucose (Gibco) and 10% fetal calf serum (Gibco) for 24 h at 37°C, 5% CO_2_. *OsCNGC13* and *OsCNGC13*-*D* were cloned into the pCI-neo vector to generate the pCI-neo-OsCNGC13 and pCI-neo-OsCNGC13-D constructs, respectively (see S3 Table for primers and cloning sites). Then the pCI-neo-OsCNGC13 and pCI-neo-OsCNGC13-D plasmid were individually co-transfected with pEGFP-N1 into HEK293 cells using the Lipofectamine 2000 Transfection Reagent (Invitrogen). Then, the transfected cells were treated with Trypsin (Gibco), centrifuged at 160g for 5 min, and kept on ice for patch-clamp recording. The cells with GFP fluorescence were selected for whole-cell recording. The method for K^+^ current patch-clamp whole-cell recording was described previously [34]. For Ca^2+^ current recordings, standard whole-cell recording techniques were applied [18]. The components of the standard Ca^2+^ pipette solution were 8 mM NaCl, 120 mM CsCl, 6.7 mM EGTA, 3.35 mM CaCl_2_, 3 mM MgCl_2_, 10 mM Hepes, 2.5 mM Mg-ATP added freshly and D-sorbito (л = 350 mosmol kg^-1^), pH 7.2 adjusted with NaOH. The standard bath solution for Ca^2+^ current recordings contains 120 mM NaCl, 10 mM CsCl, 2 mM MgCl_2_, 10 mM CaCl_2_, 10 mM Hepes and D-sorbito (л = 350 mosmol kg^-1^), pH 7.2 adjusted with NaOH. The patch-clamp recordings were conducted at about 20°C in dim light. Whole-cell currents were recorded using an Axopatch 200B amplifier (Axon Instruments).

### SEM-EDX analysis

Samples were collected and rapidly frozen in liquid nitrogen and vacuum freeze dried at -80°C for 7 d. Hand-cut sections of the freeze-dried samples were gold coated in a high-vacuum sputter coater and analyzed using a Hitachi S-3400N scanning electron microscope equipped with an energy dispersive x-ray spectrometer (EX-250; Horiba) that was interfaced with the IXRF system under the following conditions: accelerating voltage, 10 kV; takeoff angle, 35°C; collecting time of x-ray counts, 50 s; working distance between sample and detector, 15 mm. Probe measurements of pistils were made with a broad electron beam covering the whole cross-section. The total amounts of C, O, Na^+^, Mg^2+^ and Ca^2+^ were measured, and the relative amounts of Ca^2+^ were expressed as the weight fraction (Wt.%).

### Measurement of [Ca^2+^]_cyt_

For Fluo-3/AM staining, fresh pistils were excised with a razor and immediately placed in 10 mM MES buffer or 10 mM MES buffer containing 20 μM Fluo-3/AM (Molecular Probe, USA). After 5 h incubation at 4°C in the dark, the samples were washed with fresh Fluo-3/AM-free MES buffer before microscopic examination. Fluorescence from the pistils loaded with Fluo-3/AM was detected at 488 nm (excitation) and 520 nm (detection) under a laser scanning confocal microscope (ZEISS LSM 700).

For YC3.6 fluorescence observation, the *UBQ10*::*YC3*.*6* transgenic plants [38] were crossed with the wild type 9311 and the *sss1-D* mutant, respectively. The pistils of the wild type plants and the homozygous mutants in the F_2_ populations were excised with a razor and observed directly at 458 nm (excitation) and 525 nm (detection) under a laser scanning confocal microscope (ZEISS LSM 700). ZEN microscope and imaging software were used to measure the mean fluorescence intensity.

### Potassium pyroantimonate precipitation

Samples were fixed in a fixative of 2.5% glutaraldehyde / 2% potassium pyroantimonate (pH 7.6) buffered in 100 mmol/L phosphate buffer for 12 h at 4°C. Then the tissues were washed in 2% potassium pyroantimonate buffered in 100mmol/L phosphate buffer for 2 h, post fixed in 1% osmium tetraoxide / 2% potassium pyroantimonate (pH 7.6) at 4°C overnight. The postfixed tissues were washed with distilled water for 3 times with 30 min each time, followed by dehydration in a graded ethanol series and embedded in acrylic resin (London Resin Company). Ultrathin sections (70 nm) were double stained with 2% (w/v) uranyl acetate and 2.6% (w/v) lead citrate aqueous solution and examined with a transmission electron microscopy (HT7700; Hitachi).

### Histological and microscopic examination

The pistils were collected and fixed in FAA for 24 h at 4°C, dehydrated through an ethanol series, and then embedded in paraffin. For Alcian blue staining, tissue cross-sections (7 μM thickness) were cut and hydrated with an ethanol series and stained with Alcian blue and 1% Nuclear Fast Red [11] before microscopic examination (Leica DM5000B) and photographing. For the TUNEL assay, sections were cut and hydrated and treated with proteinase K in PBS. The TUNEL assay with a Dead End Fluorometric TUNEL Kit (Promega) was performed following the manufacturer’s instructions. The green fluorescence of fluorescein (TUNEL signal) and red fluorescence of propidium iodide were analyzed at 488 nm (excitation) and 520 nm (detection), and 488 nm (excitation) and 610 nm (detection), respectively, under a laser scanning confocal microscope (ZEISS LSM 700).

For plastic sections, the samples were fixed in 2.5% glutaraldehyde in a phosphate buffer for 24 h at 4°C, and dehydrated through an ethanol series; the samples were embedded in Technovit 7100 resin (Hereaus Kulzer) and polymerized at 45°C. Transverse sections of 1 μm were cut and stained with 0.1% toluidine blue O (Chroma Gesellshaft Shaud) before observation (Leica DM5000B) and photographing (Apogee Instruments).

### Accession numbers

The locus names (RAP database) for *OsCNGCs* are: *LOC_Os02g15580*, *OsCNGC1*; *LOC_Os06g33570*, *OsCNGC2*; *LOC_Os06g33610*, *OsCNGC3*; *LOC_Os03g44440*, *OsCNGC4*; *LOC_Os12g28260*, *OsCNGC5*; *LOC_Os04g55080*, *OsCNGC6*; *LOC_Os02g41710*, *OsCNGC7*; *LOC_Os12g06570*, *OsCNGC8*; *LOC_Os09g38580*, *OsCNGC9*; *LOC_Os02g54760*, *OsCNGC10*; *LOC_Os06g08850*, *OsCNGC11*; *LOC_Os02g53340*, *OsCNGC12*; *LOC_Os06g10580*, *OsCNGC13*; *LOC_Os03g55100*, *OsCNGC14*; *LOC_Os01g57370*, *OsCNGC15*; *LOC_Os05g42250*, *OsCNGC16*.

The locus names (TAIR database) for *AtCNGCs* are: *AT5G53130*, *AtCNGC1*; *AT5G15410*, *AtCNGC2*; *AT2G46430*, *AtCNGC3*; *AT5G54250*, *AtCNGC4*; *AT5G57940*, *AtCNGC5*; *AT2G23980*, *AtCNGC6*; *AT1G15990*, *AtCNGC7*; *AT1G19780*, *AtCNGC8*; *AT4G30560*, *AtCNGC9*; *AT1G01340*, *AtCNGC10*; *AT2G46440*, *AtCNGC11*; *AT2G46450*, *AtCNGC12*; *AT4G01010*, *AtCNGC13*; *AT2G24610*, *AtCNGC14*; *AT2G28260*, *AtCNGC15*; *AT3G48010*, *AtCNGC16*; *AT4G30360*, *AtCNGC17*; *AT5G14870*, *AtCNGC18*; *AT3G17690*, *AtCNGC19*; *AT3G17700*, *AtCNGC20*.

The locus names (RAP database) for the other genes in this article are: *LOC_Os01g47530*, *OsMPK20-4*; *LOC_Os03g12500*, *OsAOS2*; *LOC_Os02g43010*, *OsVPE3*.

## Supporting information

S1 Fig Distribution of seed-setting rate of the F_2_ population Q1 (n = 160). (TIF)

S2 Fig The germination of pollen grains on the stigma at 5 MAP. SEM images of the germination of pollen grains of wild type (A and B) and *sss1*-*D* (C and D) on the stigma of wild type (A and C) and *sss1*-*D* (B and D). After germination on the stigma, both the wild type and *sss1-D* pollen tubes can penetrate into the papillar cell wall. Arrow indicates the pollen tube.
(TIF)

S3 Fig Embryo sac observation at 24 HAP. (A-C) Embryo sac observation of fertilized and enlarged embryo sac of wild type (A) and *sss1-D* (B) with multi-celled globular embryo and a layer of free endosperm nuclei and the unfertilized embryo sac of *sss1-D* with unfertilized egg cell and polar nucleus (C). (D) Frequency of the fertilized embryo sac. Data are means ± SD (n = 3). **P<0.01 by the Student’s *t* test. Scale bars, 80 μm.
(TIF)

S4 Fig The *mORF1* encodes a chimeric protein OsCNGC13-D. (A) The diagrams of the OsCNGC13 and OsCNGC13-D protein structure. Numbers indicate the amino acid position. 1 to 6, transmembrane helices; P, pore-forming region; CNBD, the cyclic nucleotide binding domain. (B) Amino acid sequence alignment of OsCNGC13 and OsCNGC13-D.
(TIF)

S5 Fig Yeast two-hybrid assay. The respective constructs are shown. Note that OsCNGC13 does not interact with OsCNGC13-D. pDHB1-largeT and pXGY17-largeT were co-transformed with pDSL-Δp53 as the positive control in A and B, respectively.
(TIF)

S6 Fig BiFC assay. NY and CY stand for the N terminus and C terminus of eYFP, respectively. Note that neither OsCNGC13 nor OsCNGC13-D can form homodimer, and OsCNGC13-D cannot interact with OsCNGC13 in leaf epidermal cells of *N*. *benthamiana*. The combination of AtCNGC2 and AtCNGC4 is used as a positive control. Meanwhile, OsCNGC13-YFP and OsCNGC13-D-YFP show comparable florescence signal intensity in the plasma membrane. eYFP, enhanced yellow fluorescence protein; PM marker, PIP2;1-mCherry fusion protein; DIC, differential interference contrast; Merged, merged image of eYFP, PM marker and DIC. Scale bars, 600 μm.
(TIF)

S7 Fig The expression pattern of *OsCNGC13*. (A) Expression profiles of *OsCNGC13* from http://ricexpro.dna.affrc.go.jp/. Arrow indicates the peak expression in the pistil. (B-H) The GUS staining of primary root (B), longitudinal (C) and transverse (D) sections of primary root, the third real leaf and sheath (E), transverse sections of the third real leaf (F) and sheath (G), and spikelets at various developmental stages (H). Scale bars, 0.5 mm in (B); 50 μm in (C, D, F, and G); 1 mm in (E); 2 mm in (H).
(TIF)

S8 Fig Subcellular localization of OsCNGC13 in *N*. *benthamiana* leaf. GFP (A and B) and OsCNGC13-GFP fusion protein (C and D) transiently expressed in tobacco leaf epidermal cells (A and C) and protoplasts (B and D). Scale bars, 50 μm in (A and C); 10 μm in (B and D).
(TIF)

S9 Fig OsCNGC13 does not mediate inward K^+^ currents in HEK293 cells. Typical whole-cell recordings (A) and the average current-voltage curves (B) of steady-state inward K^+^ currents in HEK293 cells expressing *OsAKT1* (positive control) and *OsCNGC13*, respectively. The voltage protocols, as well as time and current scale bars for the recordings are shown. The data are presented as means ± SD.
(TIF)

S10 Fig Calcium precipitates in styles. (A-D) TEM images of the styles of wild type (WT) and *sss1-D* before flowering (A and B), and after pollination (C and D). (E) Style Ca^2+^ content measurement using SEM-EDX. Increased Ca^2+^ concentrations are detected in the *pORF1*::*ORF1-1* and *35S*::*ORF1-GFP-1* transgenic plants, but not in W109 after pollination. (F-K) TEM images of the styles of W109, *pORF1*::*ORF1-1* and *35S*::*ORF1-GFP-1* before flowering (F-H) and after pollination (I-K). Arrow indicates the small black calcium pyroantimonate precipitate. A few calcium precipitates could be found in the mature styles of wild type, the mutant, W109, *pORF1*::*ORF1-1* and *35S*::*ORF1-GFP-1* transgenic plants before flowering, whereas abundant calcium precipitates were detected in the styles of wild type, *pORF1*::*ORF1-1* and *35S*::*ORF1-GFP-1* transgenic plants, but not in the styles of the *sss1-D* mutant and W109, after pollination. Scale bars, 2 μm.
(TIF)

S11 Fig Style observation before flowering. (A-D) Confocal microscopy images of the style surface (A and B) and its inner structure (C and D). (E-L) Paraffin section observation of the ECM by Alcian blue staining. Transverse sections of the middle (E and F) and bottom parts (G and H) of the styles, and longitudinal sections of the middle (I and J) and bottom parts (K and L) of the styles are shown. Arrows indicate the xylem elements. (M-P) Plastic sections of the style by toluidine blue O staining. Transverse sections of the middle (M and N) and bottom parts (O and P) of the style are shown. (Q-T) TUNEL assay shows that no DNA fragmentation is observed in WT and *sss1-D* styles before flowering. Transverse sections of the middle (Q and R) and bottom parts (S and T) of the style are shown. Scale bars, 100 μm in (A-D); 50 μm in (E-T).
(TIF)

S12 Fig The phylogenetic tree of the Arabidopsis and rice CNGCs. The phylogenetic tree was constructed using full-length amino acid sequences. All the 20 Arabidopsis CNGC members (AtCNCG1 to AtCNGC20) and 16 rice CNGC members (OsCNGC1 to OsCNGC16) are shown. The bar indicates the relative divergence of the sequences examined. Numbers above the lines represent bootstrap percentages (1,000 replicates).
(TIF)

S1 Table Seed-setting rate of the reciprocal crosses. ♀, crossed as the female parents; ♂, crossed as the male parents. For each cross, the seed-setting rate was calculated using 3 independent panicles, each with at least 40 emasculated spikelets.
(PDF)

S2 Table Seed-setting rate (%), *OsCNGC13* expression levels and ovules with pollen tube (%) in several RNAi transgenic lines. Seed-setting rate (%), relative expression levels and ovules with pollen tube (%) are measured as the mean ± SD (n = 3). The relative expression level of the *OsCNGC13* gene in each transgenic plant line was determined by qRT-PCR using the primer pair 5F/5R in Fig 2D with the RNA isolated from flag leaves at heading stage. A P <0.01 was used for correlation (Pearson correlation analysis).
(PDF)

S3 Table List of primers used in this study. ^1^ The 20-bp gene-specific sequences are underlined in 20bp-pOs-Cas9-ORF1F, 20bp-pOs-Cas9-ORF1R, 20bp-pOs-Cas9-ORF3F and 20bp-pOs-Cas9-ORF3R, respectively.
(PDF)
