# Supplementary figures and images for: OsCNGC13 promotes seed-setting rate by facilitating pollen tube growth in stylar tissues
Source: PLoS Genet. 2017 Jul 14;13(7):e1006906. doi: 10.1371/journal.pgen.1006906 (PMC5533464; doi:10.1371/journal.pgen.1006906)

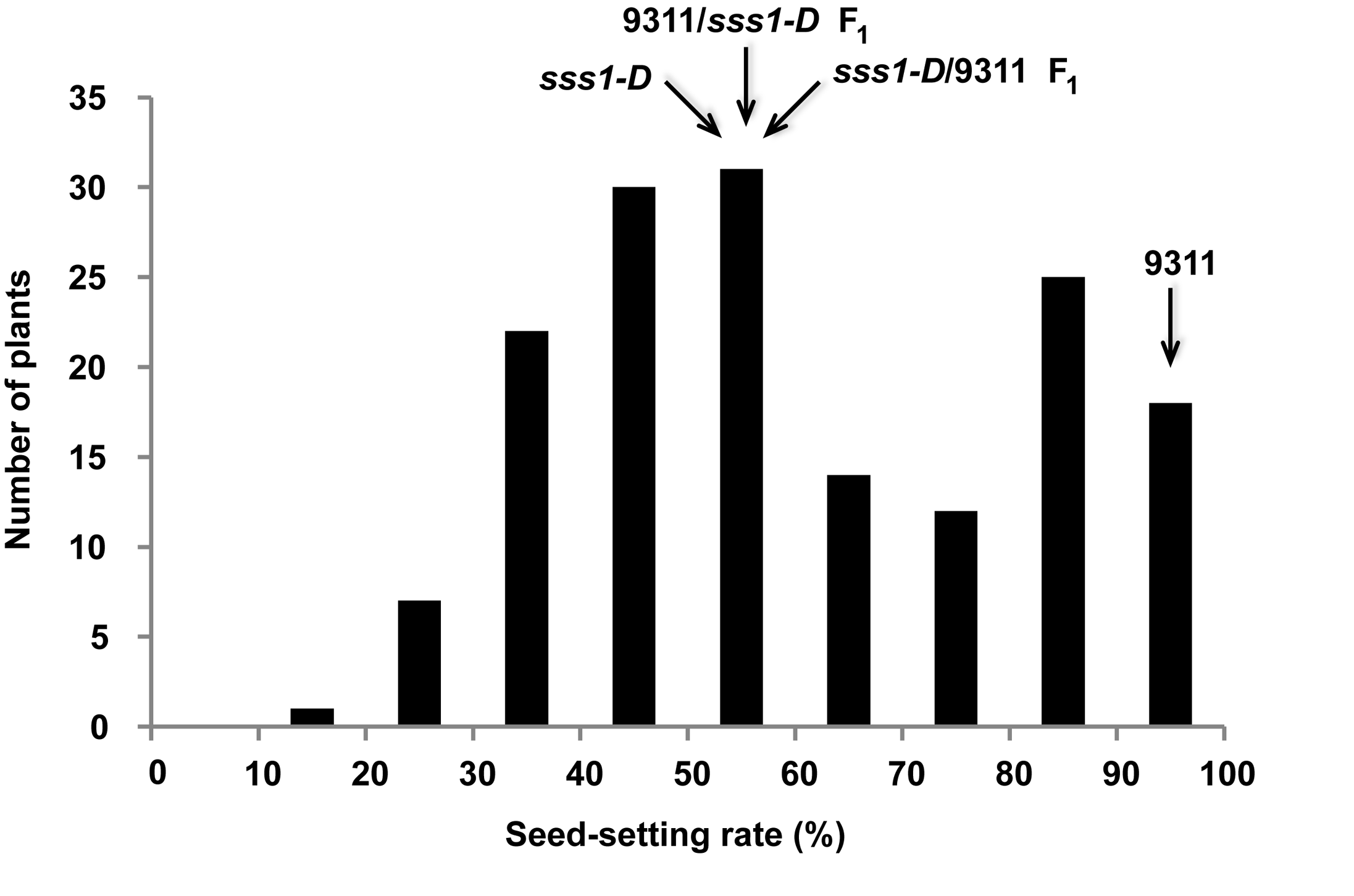

Supplement: S1 Fig — (TIF) [file pgen.1006906.s001.tif]

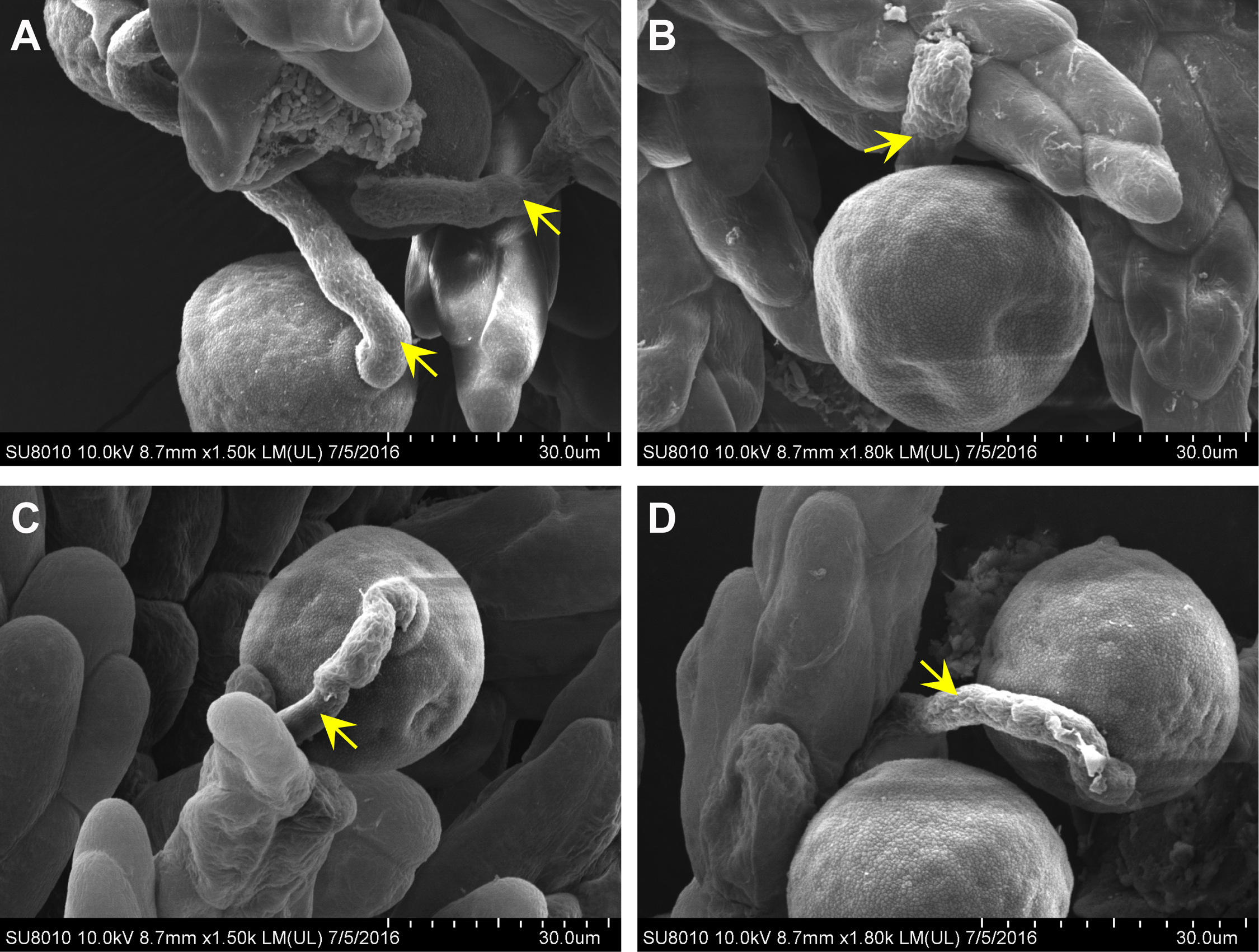

Supplement: S2 Fig — SEM images of the germination of pollen grains of wild type (A and B) and sss1-D (C and D) on the stigma of wild type (A and C) and sss1-D (B and D). After germination on the stigma, both the wild type and sss1-D pollen tubes can penetrate into the papillar cell wall. Arrow indicates the pollen tube. (TIF) [file pgen.1006906.s002.tif]

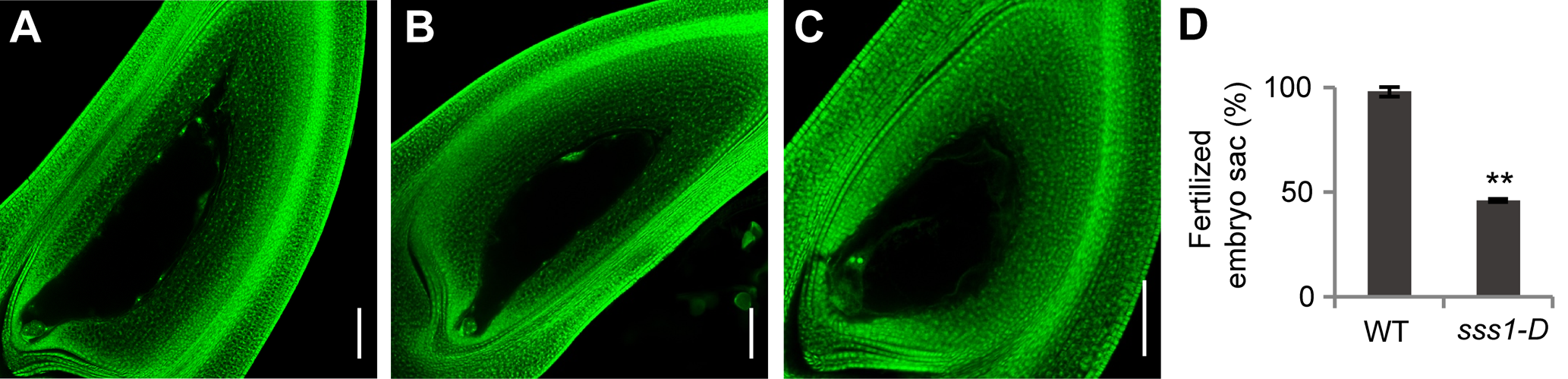

Supplement: S3 Fig — (A-C) Embryo sac observation of fertilized and enlarged embryo sac of wild type (A) and sss1-D (B) with multi-celled globular embryo and a layer of free endosperm nuclei and the unfertilized embryo sac of sss1-D with unfertilized egg cell and polar nucleus (C). (D) Frequency of the fertilized embryo sac. Data are means ± SD (n = 3). **P<0.01 by the Student’s t test. Scale bars, 80 μm. (TIF) [file pgen.1006906.s003.tif]

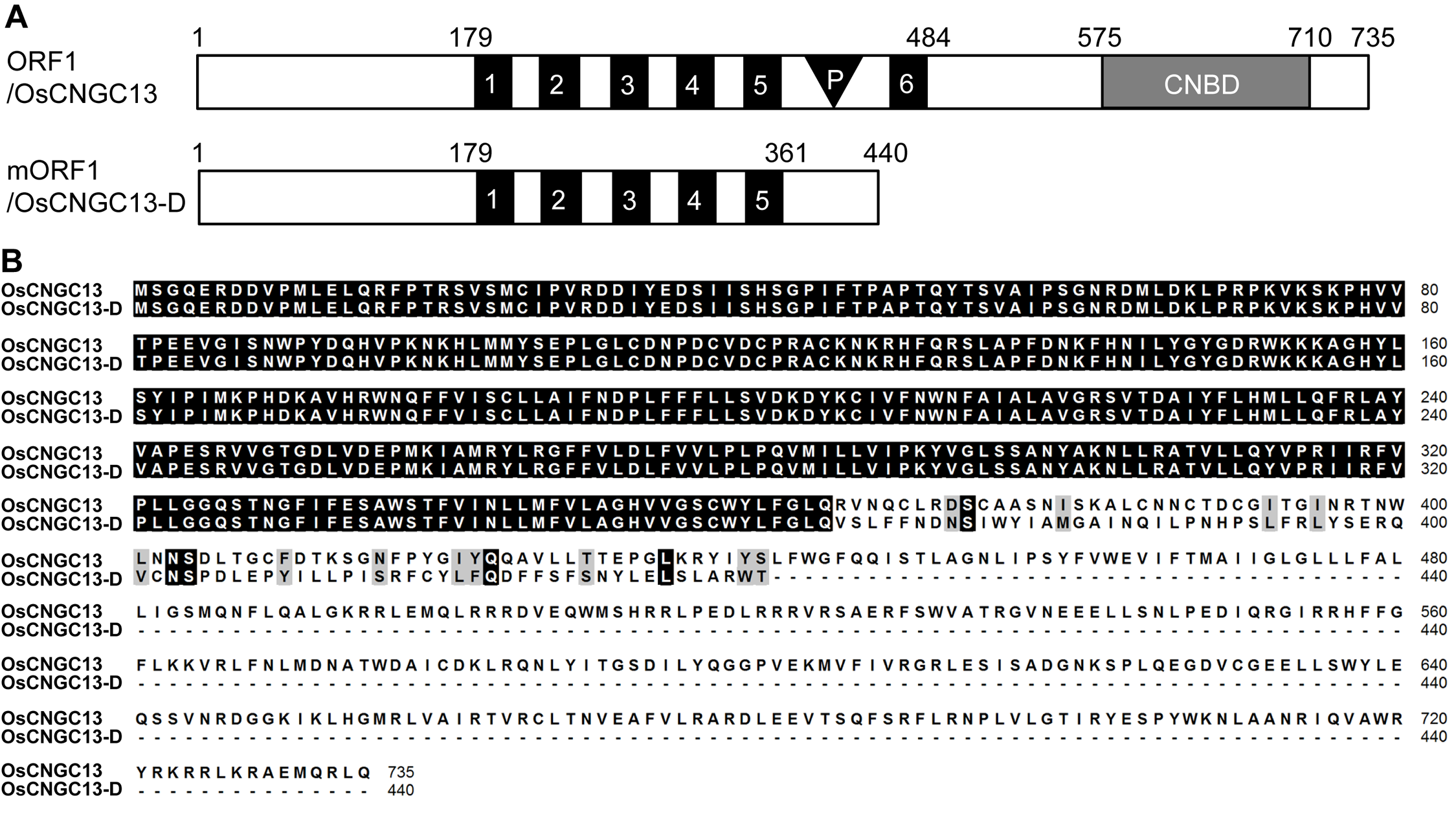

Supplement: S4 Fig — (A) The diagrams of the OsCNGC13 and OsCNGC13-D protein structure. Numbers indicate the amino acid position. 1 to 6, transmembrane helices; P, pore-forming region; CNBD, the cyclic nucleotide binding domain. (B) Amino acid sequence alignment of OsCNGC13 and OsCNGC13-D. (TIF) [file pgen.1006906.s004.tif]

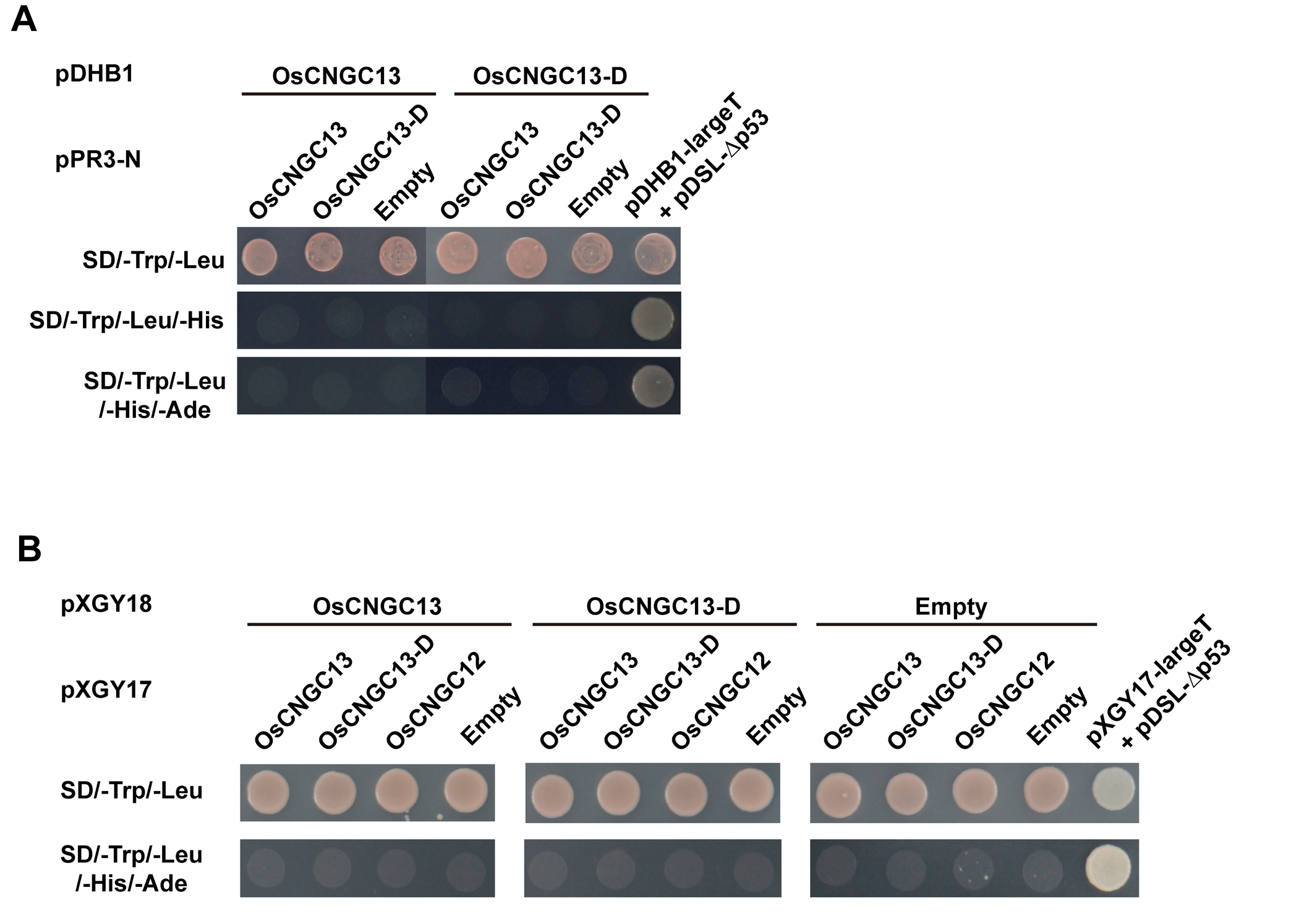

Supplement: S5 Fig — The respective constructs are shown. Note that OsCNGC13 does not interact with OsCNGC13-D. pDHB1-largeT and pXGY17-largeT were co-transformed with pDSL-Δp53 as the positive control in A and B, respectively. (TIF) [file pgen.1006906.s005.tif]

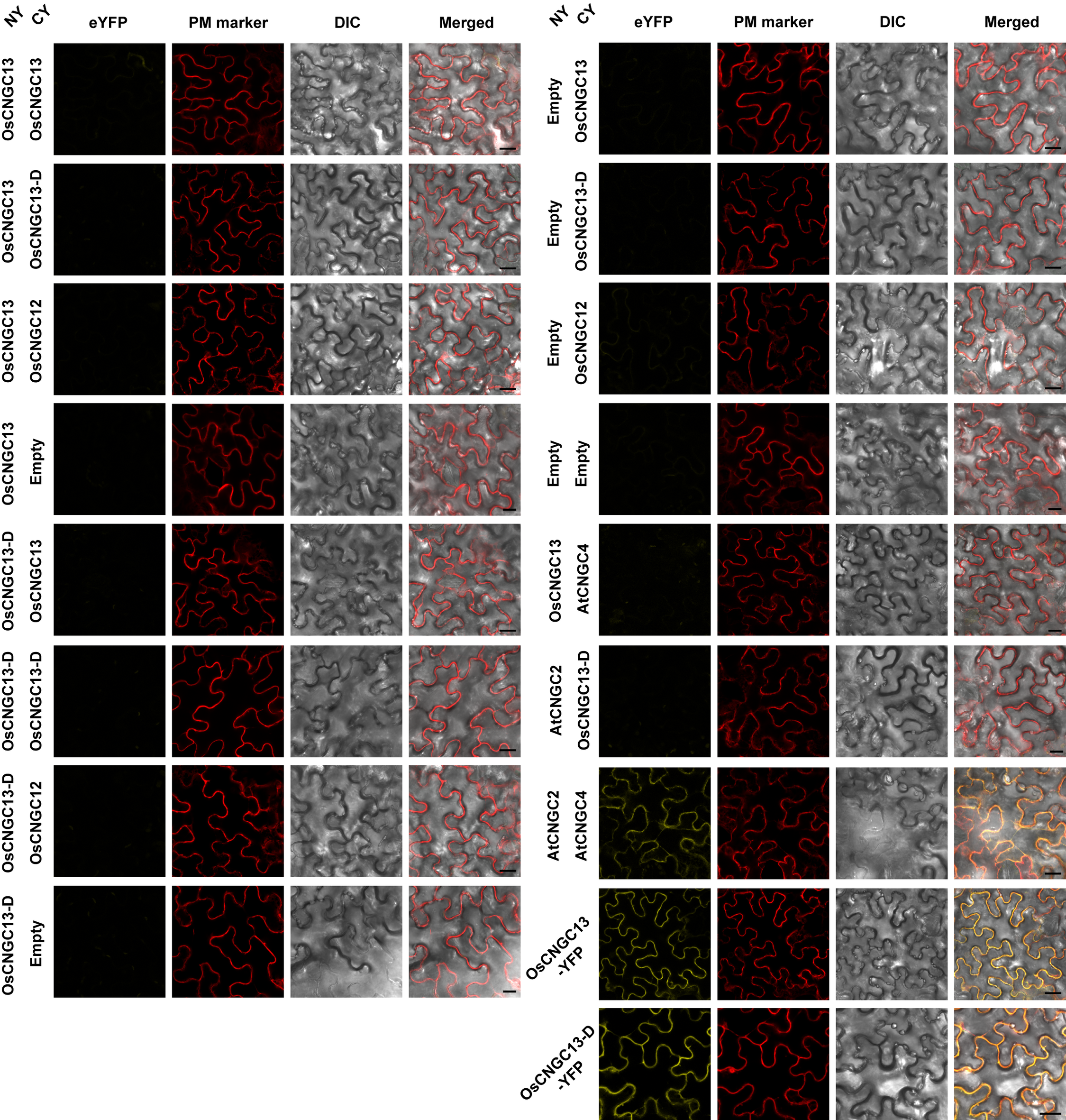

Supplement: S6 Fig — NY and CY stand for the N terminus and C terminus of eYFP, respectively. Note that neither OsCNGC13 nor OsCNGC13-D can form homodimer, and OsCNGC13-D cannot interact with OsCNGC13 in leaf epidermal cells of N. benthamiana. The combination of AtCNGC2 and AtCNGC4 is used as a positive control. Meanwhile, OsCNGC13-YFP and OsCNGC13-D-YFP show comparable florescence signal intensity in the plasma membrane. eYFP, enhanced yellow fluorescence protein; PM marker, PIP2;1-mCherry fusion protein; DIC, differential interference contrast; Merged, merged image of eYFP, PM marker and DIC. Scale bars, 600 μm. (TIF) [file pgen.1006906.s006.tif]

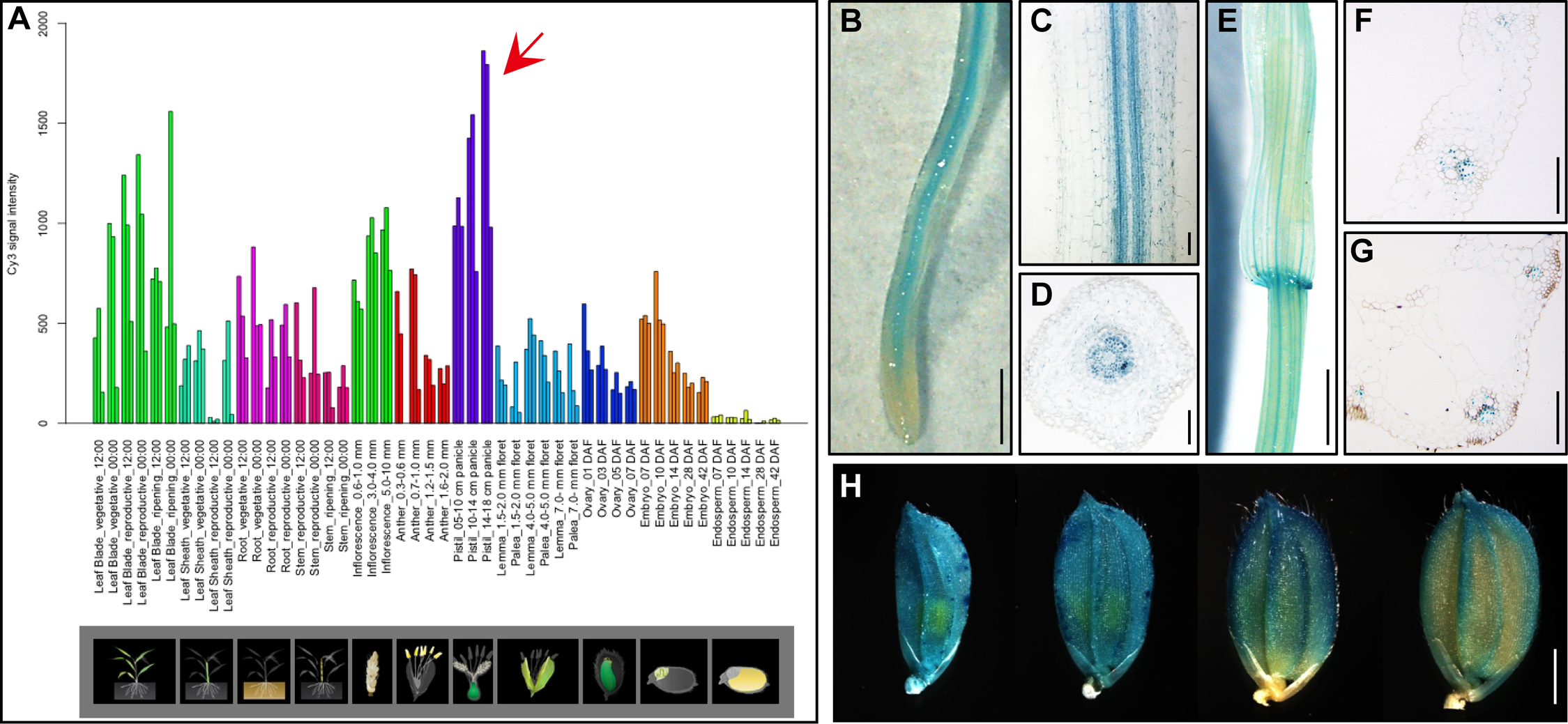

Supplement: S7 Fig — (A) Expression profiles of OsCNGC13 from http://ricexpro.dna.affrc.go.jp/. Arrow indicates the peak expression in the pistil. (B-H) The GUS staining of primary root (B), longitudinal (C) and transverse (D) sections of primary root, the third real leaf and sheath (E), transverse sections of the third real leaf (F) and sheath (G), and spikelets at various developmental stages (H). Scale bars, 0.5 mm in (B); 50 μm in (C, D, F, and G); 1 mm in (E); 2 mm in (H). (TIF) [file pgen.1006906.s007.tif]

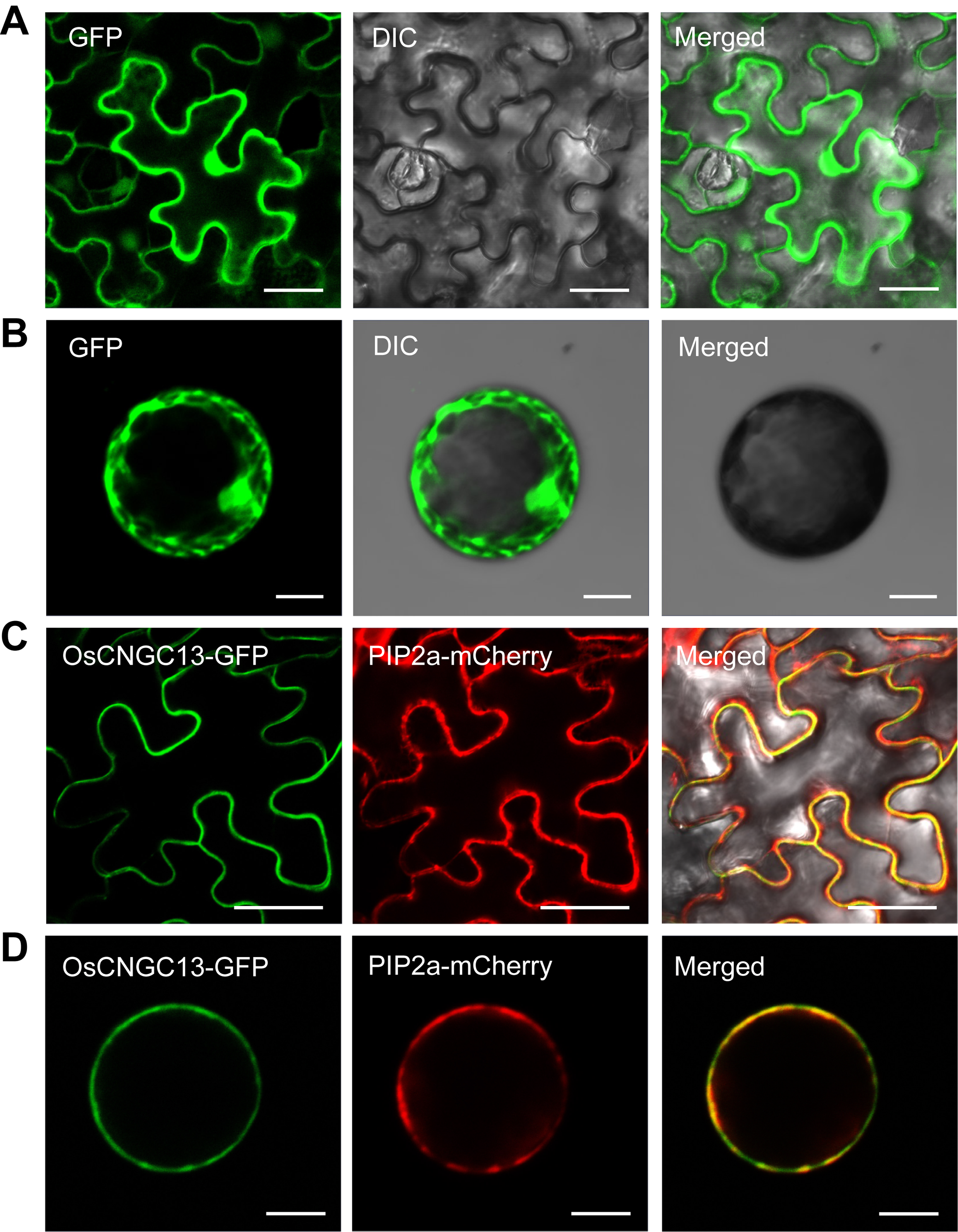

Supplement: S8 Fig — GFP (A and B) and OsCNGC13-GFP fusion protein (C and D) transiently expressed in tobacco leaf epidermal cells (A and C) and protoplasts (B and D). Scale bars, 50 μm in (A and C); 10 μm in (B and D). (TIF) [file pgen.1006906.s008.tif]

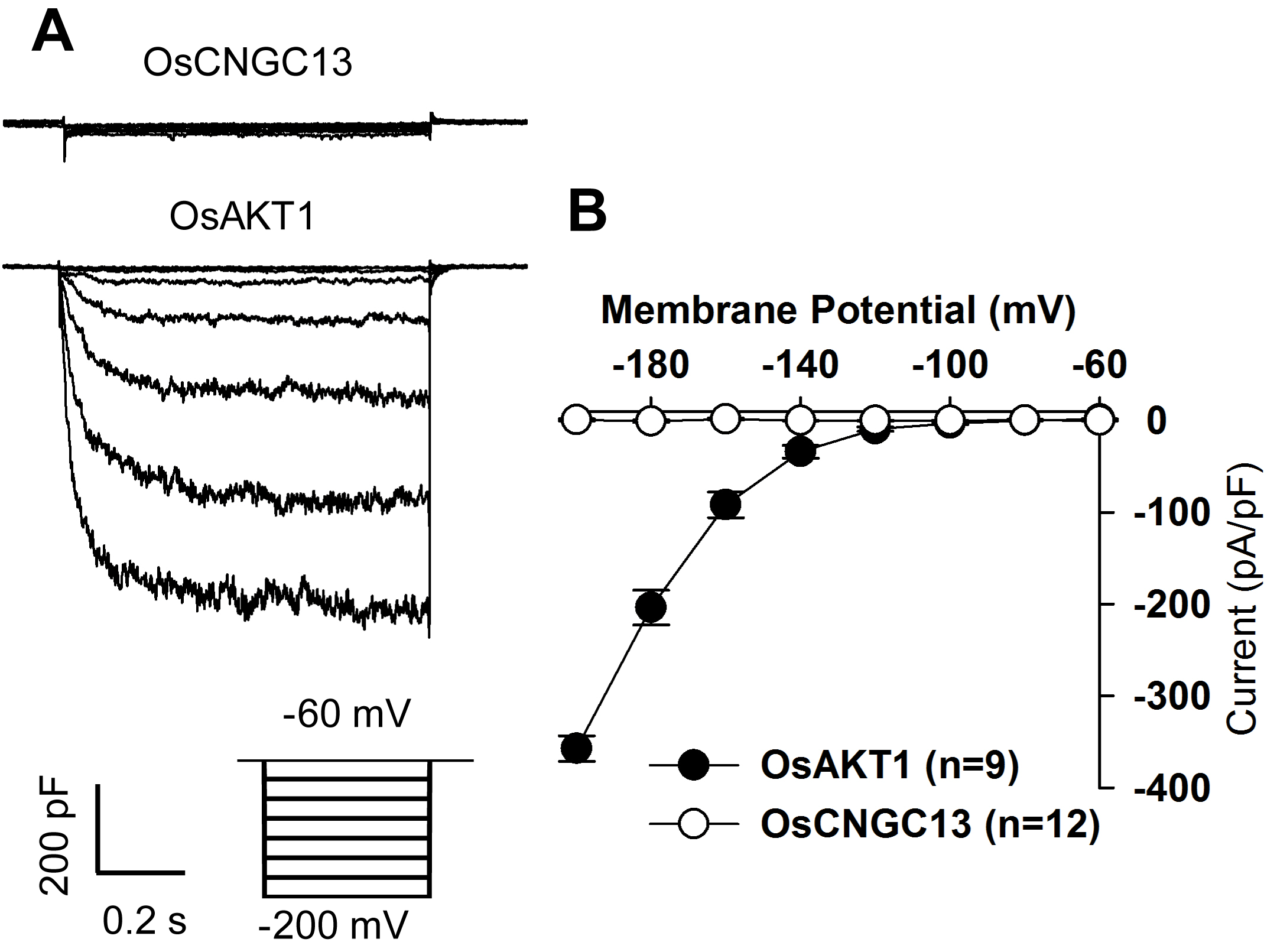

Supplement: S9 Fig — Typical whole-cell recordings (A) and the average current-voltage curves (B) of steady-state inward K+ currents in HEK293 cells expressing OsAKT1 (positive control) and OsCNGC13, respectively. The voltage protocols, as well as time and current scale bars for the recordings are shown. The data are presented as means ± SD. (TIF) [file pgen.1006906.s009.tif]

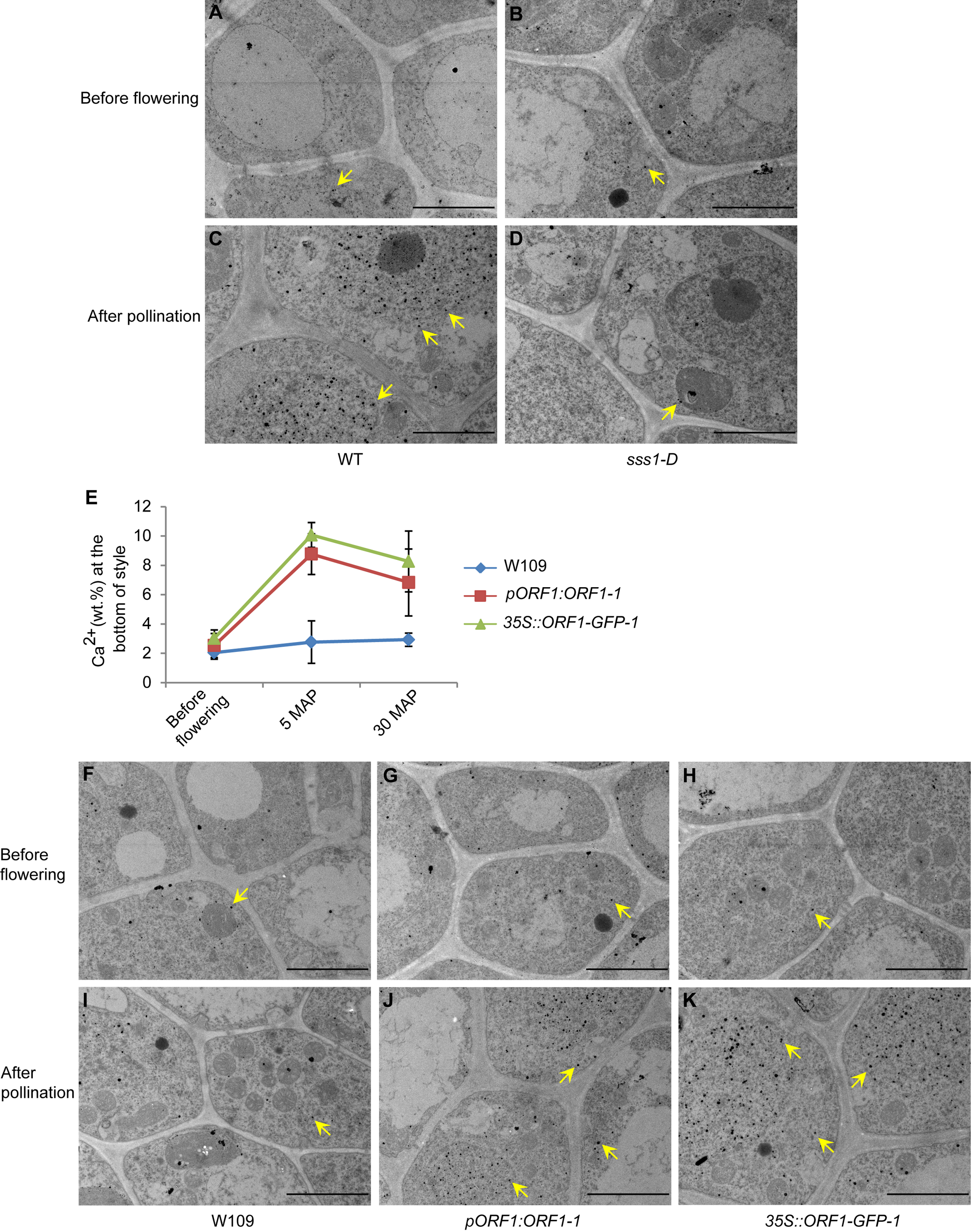

Supplement: S10 Fig — (A-D) TEM images of the styles of wild type (WT) and sss1-D before flowering (A and B), and after pollination (C and D). (E) Style Ca2+ content measurement using SEM-EDX. Increased Ca2+ concentrations are detected in the pORF1::ORF1-1 and 35S::ORF1-GFP-1 transgenic plants, but not in W109 after pollination. (F-K) TEM images of the styles of W109, pORF1::ORF1-1 and 35S::ORF1-GFP-1 before flowering (F-H) and after pollination (I-K). Arrow indicates the small black calcium pyroantimonate precipitate. A few calcium precipitates could be found in the mature styles of wild type, the mutant, W109, pORF1::ORF1-1 and 35S::ORF1-GFP-1 transgenic plants before flowering, whereas abundant calcium precipitates were detected in the styles of wild type, pORF1::ORF1-1 and 35S::ORF1-GFP-1 transgenic plants, but not in the styles of the sss1-D mutant and W109, after pollination. Scale bars, 2 μm. (TIF) [file pgen.1006906.s010.tif]

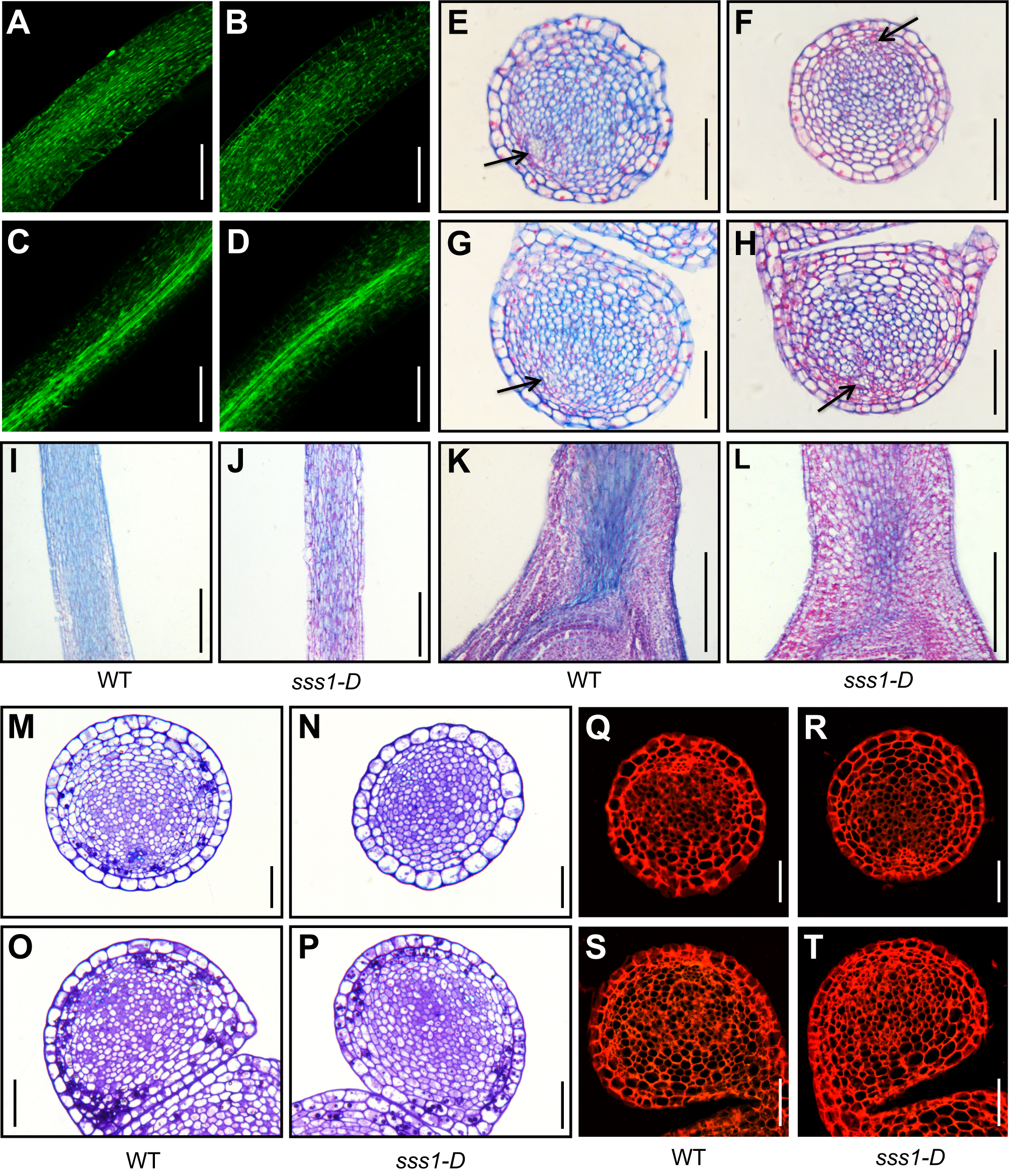

Supplement: S11 Fig — (A-D) Confocal microscopy images of the style surface (A and B) and its inner structure (C and D). (E-L) Paraffin section observation of the ECM by Alcian blue staining. Transverse sections of the middle (E and F) and bottom parts (G and H) of the styles, and longitudinal sections of the middle (I and J) and bottom parts (K and L) of the styles are shown. Arrows indicate the xylem elements. (M-P) Plastic sections of the style by toluidine blue O staining. Transverse sections of the middle (M and N) and bottom parts (O and P) of the style are shown. (Q-T) TUNEL assay shows that no DNA fragmentation is observed in WT and sss1-D styles before flowering. Transverse sections of the middle (Q and R) and bottom parts (S and T) of the style are shown. Scale bars, 100 μm in (A-D); 50 μm in (E-T). (TIF) [file pgen.1006906.s011.tif]

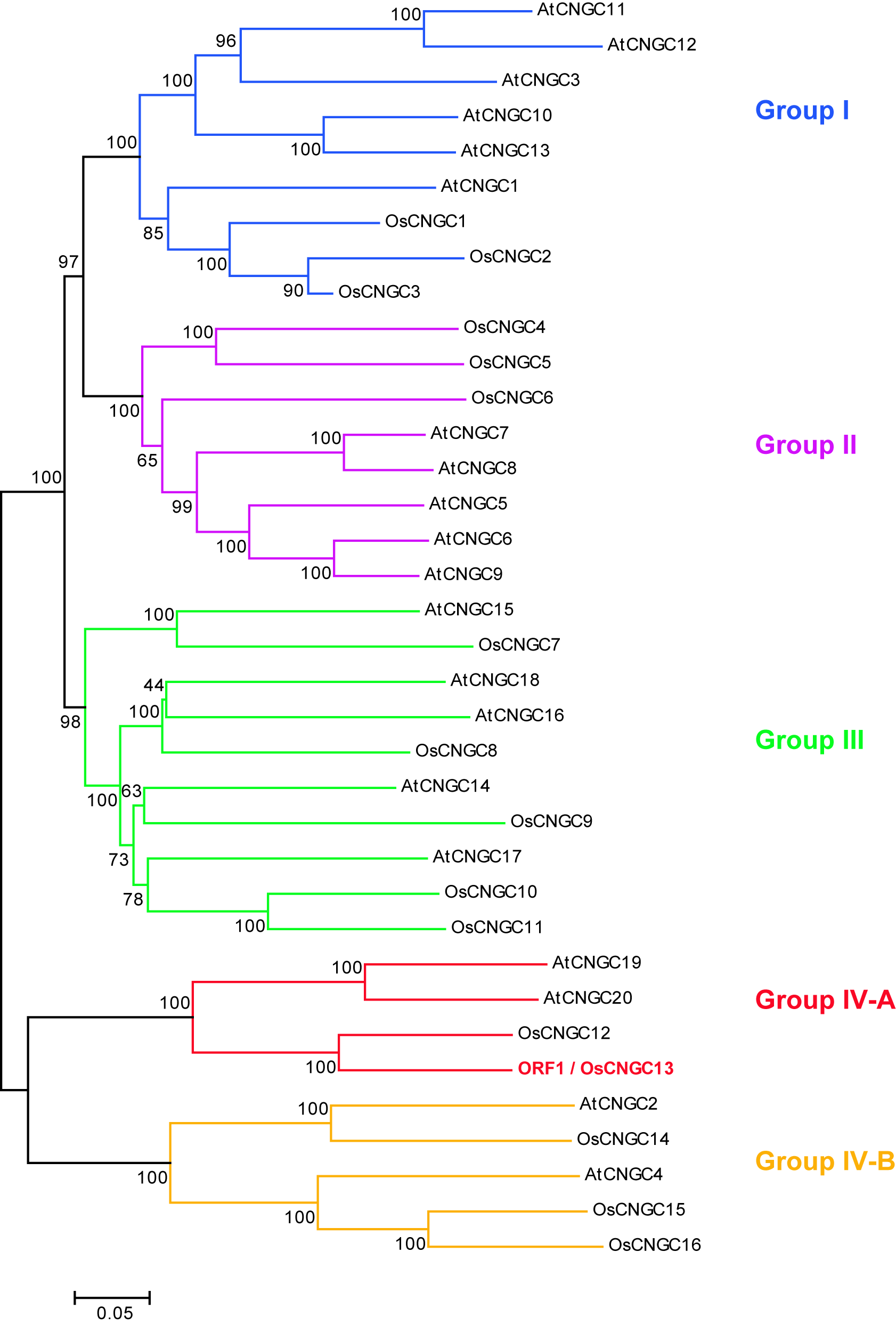

Supplement: S12 Fig — The phylogenetic tree was constructed using full-length amino acid sequences. All the 20 Arabidopsis CNGC members (AtCNCG1 to AtCNGC20) and 16 rice CNGC members (OsCNGC1 to OsCNGC16) are shown. The bar indicates the relative divergence of the sequences examined. Numbers above the lines represent bootstrap percentages (1,000 replicates). (TIF) [file pgen.1006906.s012.tif]
