# Supplementary material for: OsCNGC13 promotes seed-setting rate by facilitating pollen tube growth in stylar tissues
Source: PLoS Genet. 2017 Jul 14;13(7):e1006906. doi: 10.1371/journal.pgen.1006906 (PMC5533464; doi:10.1371/journal.pgen.1006906)
Supplement: S1 Table — ♀, crossed as the female parents; ♂, crossed as the male parents. For each cross, the seed-setting rate was calculated using 3 independent panicles, each with at least 40 emasculated spikelets. (PDF) [file pgen.1006906.s013.pdf]

S1 Table. Seed-setting rate of the reciprocal crosses.

Materials		Seed-setting rate (%)	
		♂	
		9311	*sss1-D*
♀	9311	87.7 ± 6.8	91.1 ± 5.2
	sss1-D	41.9 ± 9.4	30 ± 17.3

♀, crossed as the female parents; ♂, crossed as the male parents. For each cross, the seed-setting rate was calculated using 3 independent panicles, each with at least 40 emasculated spikelets.
