# Supplementary material for: OsCNGC13 promotes seed-setting rate by facilitating pollen tube growth in stylar tissues
Source: PLoS Genet. 2017 Jul 14;13(7):e1006906. doi: 10.1371/journal.pgen.1006906 (PMC5533464; doi:10.1371/journal.pgen.1006906)
Supplement: S2 Table — Seed-setting rate (%), relative expression levels and ovules with pollen tube (%) are measured as the mean ± SD (n = 3). The relative expression level of the OsCNGC13 gene in each transgenic plant line was determined by qRT-PCR using the primer pair 5F/5R in Fig 2D with the RNA isolated from flag leaves at heading stage. A P <0.01 was used for correlation (Pearson correlation analysis). (PDF) [file pgen.1006906.s014.pdf]

S2 Table. Seed-setting rate (%), *OsCNGC13* expression levels and ovules with pollen tube (%) in several RNAi transgenic lines.

Background	Lines	Seed-setting rate (%)	Relative mRNA level	Correlation coefficient	Ovules with pollen tube (%)	Correlation coefficient
Kitaake	WT	98.04 ± 3.396	1 ± 0.006	0.939**	94.44 ± 1.571	0.909**
Kitaake ORF1-RNAi lines	WT$^{ORF1\text{-RNAi}}$-1	62.89 ± 8.460	0.05 ± 0.001		54.44 ± 5.666	
	WT$^{ORF1\text{-RNAi}}$-3	64.73 ± 1.681	0.16 ± 0.002		57.78 ± 1.571	
	WT$^{ORF1\text{-RNAi}}$-4	80.96 ± 3.511	0.65 ± 0.009		80.00 ± 7.201	
	WT$^{ORF1\text{-RNAi}}$-5	59.85 ± 7.387	0.03 ± 0.001		57.78 ± 5.666	
	WT$^{ORF1\text{-RNAi}}$-7	67.63 ± 1.673	0.17 ± 0.005		61.11 ± 6.285	
	WT$^{ORF1\text{-RNAi}}$-8	66.80 ± 3.717	0.05 ± 0.003		58.89 ± 6.285	
	WT$^{ORF1\text{-RNAi}}$-9	77.33 ± 13.31	0.56 ± 0.005		82.22 ± 1.571	
	WT$^{ORF1\text{-RNAi}}$-10	67.85 ± 9.425	0.07 ± 0.001		53.33 ± 9.425	
	WT$^{ORF1\text{-RNAi}}$-11	82.54 ± 4.864	0.82 ± 0.013		87.78 ± 5.443	
	WT$^{ORF1\text{-RNAi}}$-12	57.04 ± 11.4	0.08 ± 0.006		61.11 ± 1.571	
	WT$^{ORF1\text{-RNAi}}$-13	62.27 ± 3.642	0.03 ± 0.003		57.78 ± 1.571	
	WT$^{ORF1\text{-RNAi}}$-14	66.67 ± 5.774	0.07 ± 0.003		66.67 ± 2.722	
	WT$^{ORF1\text{-RNAi}}$-15	56.27 ± 4.194	0.07 ± 0.001		47.78 ± 7.573	
	WT$^{ORF1\text{-RNAi}}$-16	57.62 ± 5.487	0.06 ± 0.004		61.11 ± 5.666	
	WT$^{ORF1\text{-RNAi}}$-29	63.74 ± 7.705	0.15 ± 0.001		57.78 ± 3.721	

Seed-setting rate (%), relative expression levels and ovules with pollen tube (%) are measured as the mean ± SD (n=3). The relative expression level of the *OsCNGC13* gene in each transgenic plant line was determined by qRT-PCR using the primer pair 5F/5R in Fig 2D with the RNA isolated from flag leaves at heading stage. A P <0.01 was used for correlation (Pearson correlation analysis).
