# Supplementary material for: OsCNGC13 promotes seed-setting rate by facilitating pollen tube growth in stylar tissues
Source: PLoS Genet. 2017 Jul 14;13(7):e1006906. doi: 10.1371/journal.pgen.1006906 (PMC5533464; doi:10.1371/journal.pgen.1006906)
Supplement: S3 Table — 1 The 20-bp gene-specific sequences are underlined in 20bp-pOs-Cas9-ORF1F, 20bp-pOs-Cas9-ORF1R, 20bp-pOs-Cas9-ORF3F and 20bp-pOs-Cas9-ORF3R, respectively. (PDF) [file pgen.1006906.s015.pdf]

S3 Table. List of primers used in this study.

Mapping primers			
Primer name	Forward sequence (5'-3')	Reverse sequence (5'-3')	
Indel			
6-26	CTCAACGTTGACACCTCGTG	TCCTCCATCGAGCAGTATCA	
Q-7	CATCCCAAACTACCAACCAA	GAGATGTGACGAGGGAAGAG	
Q-17	GACACGTTTAGCTGCGTAGTT	CCTTCAATGGTCGTATCTTCT	
Q-26	TACTAGATGGGATGAAAATCA	GAATAATGCCTCACCTGTGC	
Y-6	TAGGTGGATCTATTGTATTTA	CAATTCTCTTATACTTCAACA	
Y-13	TCCGATGCTTCAATCAACAGTC	GGTTTGGGAGGATTGGGAAT	
Y-14	AAATAAAAAAAGAAGGGGCT	TTCAATGAGAAGTTGGTGGT	
Y-80	GCTGCTACTACCTCTGCCTCTTCC	TCAATCGATCCATCCATCTCTCC	
dCAPS			Restriction enzyme
Q-20	GTCAGTAGCAGATC	AACGTTGGTGCATAGCGGAC	BglII
Primers for confirming the fragment inversion			
Primer name	Primer sequence (5' to 3')		
1F	ACAAGAATCCCGTAGACAT		
1R	CATTAGCACGTTGGACATA		
2F	CATACAATGACCCACTCCTAAC		
2R	CTTCATCATGCTCCTCTTCC		
3F	TCTTAGCAGCGAGATGCAGTTCC		
3R	TGTGCCGCACGCAATGGT		
4F	AGATTGTATTCAGAAAGGCAGGTC		
4R	TGGGTGTAGATGTACGAGGAGC		
Quantitative PCR Primers			
Primer name	Primer sequence (5' to 3')		
5F	AGTAGTGGGAACTGGAGATTTGG		
5R	CATCACCTGAGGGAGTGGAAG		
6F	TGCTAACCACAGAACCTGGACTT		
6R	GGACCCTATGAGTAATGCGAAAA		
7F	TGGGCATGTCGTTGGTTCA		
7R	ACAGGCTTGGGTGGTTGG		
Primer name	Forward sequence (5'-3')	Reverse sequence (5'-3')	
qRT-ORF2	AGACAGAGACAGGGAGATGTTGC	AGCAGCAGAGGATGAATGGTTCG	
qRT-ORF3	CGCCGAGAACGACAAGAT	CGACACGCGGTCCAGC	
OsMPK20-4	GATTCCAGGTAGAACAGGAC	GCTCTCTTTCTGAGCTATCC	
OsAOS2	CAATACGTGTACTGGTCGAATGG	AAGGTGTCGTACCGGAGGAA	
OsVPE3	CGGTAACTACAGGCACCAGGC	GTGACTTCGTCTCCAGTGTAATCC	
Ubq	GCTCCGTGGCGGTATCAT	CGGCAGTTGACAGCCCTAG	
3' RACE gene-specific primers			
Primer name	Primer sequence (5' to 3')		
RACE-1	TAGTGGGAACTGGAGATTTGGTTGATGAG		

RACE-2	GGGCATGTCGTTGGTTCATGTTGGTA	
RACE-3	TCAGATACTTCCCAACCACCCAAGCC	
Binary vector construction primers		
Primer name	Primer sequence (5' to 3')	Restriction enzyme
pORF1-1305-EcoRI-F	ATT**GAATTC**CATCCATTTTAGGGTTCCAC	EcoRI
pORF1-1305-KpnI-R	ATT**GGTACC**TTCCTTACACGGAACCCAAC	KpnI
ORF1-1305-BamHI-F	AGGTACCCGG**GGATCC**ATGTCTGGCCAAGAGAGAGA	BamHI
ORF1-1305-XbaI-R	GCAGGTCGAC**TCTAGA**CTATTGCAACCTTTGCATCT	XbaI
ORF1-1305-GFP-SpeI-F	ATT**ACTAGT**ATGTCTGGCCAAGAGAGAG	SpeI
ORF1-1305-GFP-BamHI-R	AAT**GGATCC**TTGCAACCTTTGCATC	BamHI
gORF3-1305-EcoRI-F	CCATGATTAC**GAATTC**TTCCTACCCATCATAAACCAT	EcoRI
gORF3-1305-EcoRI-R	AAAATGGATG**GAATTC**TACCCGAACCATTCATCCT	EcoRI
ORF3-1305-GFP-SpeI-F	ATT**ACTAGT**ATGGATTTCGGCGACGAACC	SpeI
ORF3-1305-GFP-BamHI-R	AAT**GGATCC**GGGGTGGTGGCCATGGGCGG	BamHI
OsCNGC13-D-1305-BamHI-F	AGGTACCCGG**GGATCC**ATGTCTGGCCAAGAGAGAGA	BamHI
OsCNGC13-D -1305-XbaI-R	GCAGGTCGAC**TCTAGA**TTATGTCCAACGTGCTAATG	XbaI
20bp-pOs-Cas9-ORF1F [1]	AGATGATCCGTGGCAGACTCAATCATATCCCACAGGT TTTAGAGCTATGC	BsaI
20bp-pOs-Cas9-ORF1R	GCATAGCTCTAAAACCTGTGGGATATGATTGAGTCTG CCACGGATCATCT	BsaI
20bp-pOs-Cas9-ORF3F	AGATGATCCGTGGCACCCGCTGGTTCTCGTCCGGGG TTTTAGAGCTATGC	BsaI
20bp-pOs-Cas9-ORF3R	GCATAGCTCTAAAACCCCGGACGAGAACCAGCGGGT GCCACGGATCATCT	BsaI
ORF1RNAi-B-K	AAT **GGATCC GGTACC** TTGAACAACTCAGACTTAAC	BamHI, KpnI
ORF1RNAi-M-S	AAT **ACGCGT GAGCTC** AGCCGGACCTTCTTAAGGAA	MluI, SacI
pOsCNGC13-1305-GUS-EcoRI-F	CCATGATTAC**GAATTC**CATCCATTTTAGGGTTCCAC	EcoRI
pOsCNGC13-1305-GUS-NcoI-R	CTCAGATCTA**CCATGG**TTCCTTACACGGAACCCAAC	NcoI
In situ hybridization primers		
OsCNGC13-pGEM-T-F	ATGCTAGAGCTACAAAGATT	
OsCNGC13-pGEM-T-R	CTGGTCATAAGGCCAGTTAC	
Subcellular localization in rice protoplasts primer		
OsCNGC13-pA7-GFP-XhoI-F	ACGAACGATA**CTCGAG**ATGTCTGGCCAAGAGAGAGA	XhoI
OsCNGC13-pA7-GFP-SpeI-R	GCTCACCATC**ACTAG** TTGCAACCTTTGCATCTCAG	SpeI
Yeast two-hybrid vector construction primers		
OsCNGC13-pDHB1-NcoI-F	GGCCAGGCCT**CCATGG**ATGTCTGGCCAAGAGAGAGA	NcoI
OsCNGC13-pDHB1-NcoI-R	CCAAGATATA**CCATGG**TTGCAACCTTTGCATCTCAG	NcoI
OsCNGC13-D-pDHB1-NcoI-F	GGCCAGGCCT**CCATGG**ATGTCTGGCCAAGAGAGAGA	NcoI

OsCNGC13-D-pDHB1-NcoI-R	CCAAGATATA**CCATGG**TGTCCAACGTGCTAATGACA	NcoI
OsCNGC13-pPR3-N-BamHI-F	AGATTACGCT**GGATCC**ATGTCTGGCCAAGAGAGAGA	BamHI
OsCNGC13-pPR3-N-BamHI-R	ACCACTGCTT**GGATCC**TTGCAACCTTTGCATCTCAG	BamHI
OsCNGC13-D-pPR3-N-BamHI-F	AGATTACGCT**GGATCC**ATGTCTGGCCAAGAGAGAGA	BamHI
OsCNGC13-D-pPR3-N-BamHI-R	ACCACTGCTT**GGATCC**TGTCCAACGTGCTAATGACA	BamHI
BiFC assay primers		
OsCNGC13-p2Y-N- PacI-F	CATTTACGAACGATAG**TTAATTAA**ATGTCTGGCCAAGAGAGAGA	PacI
OsCNGC13-p2Y-N- SpeI-R	CACTGCCACCTCCTCC**ACTAGT**TTGCAACCTTTGCATCTCAG	SpeI
OsCNGC13-D-p2Y-C- PacI-F	CATTTACGAACGATAG**TTAATTAA**ATGTCTGGCCAAGAGAGAGA	PacI
OsCNGC13-D-p2Y-C- SpeI-R	CACTGCCACCTCCTCC**ACTAGT**TGTCCAACGTGCTAATGACA	SpeI
Primers used in patch-clamping recordings from HEK293 cells		
OsCNGC13-pCI-neo-EcoRI-F	CTAGCCTCGA**GAATTC**ATGTCTGGCCAAGAGAGAGA	EcoRI
OsCNGC13-pCI-neo-XbaI-R	CCGGGTCGAC**TCTAGA**CTATTGCAACCTTTGCATCT	XbaI
OsCNGC13-D-pCI-neo-EcoRI-F	CTAGCCTCGA**GAATTC**ATGTCTGGCCAAGAGAGAGA	EcoRI
OsCNGC13-D-pCI-neo-XbaI-R	CCGGGTCGAC**TCTAGA**TTATGTCCAACGTGCTAATG	XbaI

[1] The 20-bp gene-specific sequences are underlined in 20bp-pOs-Cas9-ORF1F, 20bp-pOs-Cas9-ORF1R, 20bp-pOs-Cas9-ORF3F and 20bp-pOs-Cas9-ORF3R, respectively.
